# Multiscale relevance and informative encoding in neuronal spike trains

**DOI:** 10.1007/s10827-020-00740-x

**Published:** 2020-01-28

**Authors:** Ryan John Cubero, Matteo Marsili, Yasser Roudi

**Affiliations:** 1 Kavli Institute for Systems Neuroscience and Centre for Neural Computation, Norwegian University of Science and Technology (NTNU), Trondheim, Norway; 2 The Abdus Salam International Center for Theoretical Physics, Trieste, Italy; 3 Scuola Internazionale Superiore di Studi Avanzati, Trieste, Italy; 4 IST Austria, Klosterneuburg, Austria; 5 Istituto Nazionale di Fisica Nucleare (INFN), Sezione di Trieste, Italy

**Keywords:** Time series analysis, Multiple time scale analysis, Spike train data, Information theory, Bayesian decoding

## Abstract

Neuronal responses to complex stimuli and tasks can encompass a wide range of time scales. Understanding these responses requires measures that characterize how the information on these response patterns are represented across multiple temporal resolutions. In this paper we propose a metric – which we call multiscale relevance (MSR) – to capture the dynamical variability of the activity of single neurons across different time scales. The MSR is a non-parametric, fully featureless indicator in that it uses only the time stamps of the firing activity without resorting to any *a priori* covariate or invoking any specific structure in the tuning curve for neural activity. When applied to neural data from the mEC and from the ADn and PoS regions of freely-behaving rodents, we found that neurons having low MSR tend to have low mutual information and low firing sparsity across the correlates that are believed to be encoded by the region of the brain where the recordings were made. In addition, neurons with high MSR contain significant information on spatial navigation and allow to decode spatial position or head direction as efficiently as those neurons whose firing activity has high mutual information with the covariate to be decoded and significantly better than the set of neurons with high local variations in their interspike intervals. Given these results, we propose that the MSR can be used as a measure to rank and select neurons for their information content without the need to appeal to any *a priori* covariate.

**Electronic supplementary material:**

The online version of this article (10.1007/s10827-020-00740-x) contains supplementary material, which is available to authorized users.

## Introduction

Much of the progress in understanding how the brain processes information has been made by identifying firing patterns of individual neurons that correlate significantly with the variations in the stimuli and the behaviors. These approaches have led to e.g. the discovery of V1 cells in the primary visual cortex (Hubel and Wiesel 1959), the A1 cells in the auditory cortex (Merzenich et al. 1975), the head direction cells in the anterodorsal nucleus (ADn) of the thalamus (Taube et al. 1990; Taube 1995), the place cells in the hippocampus (O’Keefe and Dostrovsky 1971) and more recently, the grid cells (Hafting et al. 2005) and speed cells (Kropff et al. 2015) in the medial entorhinal cortex (mEC). Such and subsequent studies have selected neurons based on imposed structural assumptions on the tuning profile of neurons with respect to an external correlate.

However, the organization of the brain is hardly this simple and intuitive. For instance, recent developments in understanding the spatial representation in the mEC have taught us that such approaches has its limits: First, neurons may break the symmetries of the tuning curves when representing navigational information through shearing (Stensola et al. 2015), field-to-field variability or simply by the constraints of the environment (Krupic et al. 2015). Second, the same neuron may respond to a combination of different behavioral covariates, such as position, head direction (HD) and speed in spatial navigation (Sargolini et al. 2006; Hardcastle et al. 2017). Finally, and most importantly, neurons may encode a particular behavior in ways that are unknown to the experimenter and that are not related to covariates typically used or to *a priori* features.

Under such circumstances, one can still make progress by focusing on the temporal structure of neural firing. Variations present in the spikes offer neurons with a large capacity for information transmission (Stein 1967; Rieke et al. 1993; Stein et al. 2005). Recently, it has been shown that the variations found in the relative timing of spikes carry relevant and decodable information about the behavioral task, even when the neuron’s firing rate did not increase upon stimulus presentation and decision onset (Insanally et al. 2019). These variations, as captured, for example, by metrics describing the interspike intervals, have been shown to be different for functionally distinct neurons in the cortex (Shinomoto et al. 2003, 2005, 2009) and have been utilized to classify neurons in the subiculum (Sharp and Green 1994) and in the mEC (Latuske et al. 2015; Ebbesen et al. 2016). However, such measures of variations are either very local or hardly take into account the temporal dependencies and time scales of natural stimuli that lead or contribute to the activity of the neurons.

Here, we propose a novel non-parametric, model-free method for characterizing the dynamical variability of neural spikes across different time scales and consequently, for selecting relevant neurons – i.e. neurons whose response patterns represent information about the task or stimuli – that *does not require knowledge of external correlates*. This featureless selection is done by identifying neurons that have broad and non-trivial distribution of spike frequencies across a broad range of time scales. The proposed measure – called *Multiscale Relevance* (MSR) – allows an experimenter to rank the neurons according to their information content and relevance to the behavior probed in the experiment. The theoretical arguments that lead to the definition of MSR are laid out in a number of recent publications on efficient representations (Marsili et al. 2013; Haimovici and Marsili 2015; Cubero et al. 2019); see also Battistin et al. (2017) for a concise review of this theoretical work. These arguments have been shown to be useful in characterizing the efficiency of representations in deep neuronal networks (Song et al. 2018) and in Minimum Description Length codes (Cubero et al. 2018), as well as for identifying relevant sites in proteins (Grigolon et al. 2016). The aim of this paper is to show that these arguments can also be used for studying neural representations by applying it to real and synthetic neural data.

We illustrate the method by applying it to data on spatial navigation of freely roaming rodents in Stensola et al. (2012) and Peyrache et al. (2015), that report the neural activities of 65 neurons simultaneously recorded from the medial Entorhinal Cortex (mEC), and of 746 neurons in the Anterodorsal thalamic nucleus (ADn) and Post-Subiculum (PoS), respectively. In all cases, we find that neurons with low MSR also coincide with those that contain no information on covariates involved in navigation, but that the opposite is not true. We find that some neurons with high MSR also contain significant information for spatial navigation, some relative to position, some to HD but often on both space and HD. These findings corroborate the recent conjecture of multiplexed coding (Panzeri et al. 2010) both in the mEC (Hardcastle et al. 2017), the thalamus (Mease et al. 2017) and the subiculum (Lederberger et al. 2018). We observe that MSR correlates to different degrees with different measures that have been introduced to characterize specific neurons. More specifically, we find strong correlation between MSR and measures of sparse representations of external correlates. Furthermore, we show that the neurons in mEC with highest MSR have spike patterns that allow a downstream decoder “neuron” to discern the organism’s state in the environment. Indeed, the top most relevant neurons (RNs), according to MSR, decode spatial position (or HD) just as well as the top most spatially (or HD) informative neurons (INs). In addition, we find that this decoding efficiency can not solely be due to local variations in the interspike intervals Shinomoto et al. (2003, 2005). Emphasizing again that the MSR does not rely on any information about space or HD and is calculated only from the timing on spikes, the correlation with spatial or HD information suggests a role for MSR as an unsupervised method for focusing on information-rich neurons without knowing *a priori* what covariate(s) those neurons represent.

## Multiscale relevance

Consider a neuron whose activity is observed up to a time *t*_*obs*_. This can be one of a population of *N* simultaneously recorded neurons in the same experiment. The activity of this neuron is recorded and stamped by the spike times {*t*_1_,…,*t*_*M*_} where *t*_1_ < *t*_2_ < … ≤ *t*_*M*_ ≤ *t*_*obs*_ and *M* is the total number of observed spikes. By discretizing the time into *T* bins of duration Δ*t*, a spike count code, {*k*_1_,*k*_2_,…,*k*_*T*_}, can be constructed where *k*_*s*_ denotes the number of spikes recorded from the neuron in the *s*^th^ time bin *B*_*s*_ = [(*s* − 1)Δ*t*,*s*Δ*t*) (*s* = 1,2,…,*T*).

Fixing Δ*t* allows us to probe the neural activity at a fixed time scale. Yet, rather than using Δ*t* to measure time resolution, we adopt an information theoretic measure, given by

1 $$ H[s] = -\sum\limits_{s = 1}^{T} \frac{k_{s}}{M} \log_{M} \frac{k_{s}}{M},  $$

where $\log _{M}(\cdot )=\log (\cdot )/\log M$ indicates logarithm base *M* (in units of *M* ats). Considering *k*_*s*_/*M* as the probability that the neuron fires in the bin *B*_*s*_, this has the form of a Shannon entropy (Cover and Thomas 2012). This corresponds to the amount of information that one gains on the timing of a randomly chosen spike by knowing the index *s* of the bin it belongs to.^1^

We argue that *H*[*s*] provides an intrinsic measure of resolution, contrary to Δ*t* which refers to particular time scales that may vary across neurons. For example, there is a value Δ*t*_−_ such that for all Δ*t* ≤Δ*t*_−_, all time bins either contain a single spike or none, i.e. *k*_*s*_ = 0,1 for all *s*. All these values of Δ*t* correspond to the same value of the intrinsic resolution *H*[*s*] = 1. Likewise, there may be a value Δ*t*_+_ such that for all Δ*t* ≥Δ*t*_+_, all spikes of the neuron fall in the same bin. All Δ*t* ≥Δ*t*_+_ then correspond to the same value *H*[*s*] = 0 of the resolution, as defined here. In other words, *H*[*s*] captures resolution on a scale that is fixed by the available data.

Given a resolution *H*[*s*] (corresponding to a given Δ*t*), we can now turn to characterize the dynamic response of the neuron. The only way in which the dynamic state of the neuron in bin *s* can be distinguished from that in bin $s^{\prime }$ is by its activity. If the number of spikes in the two bins is the same ($k_{s}= k_{s^{\prime }}$) there is no way to distinguish the dynamic state of the neuron in the two bins, at that resolution;^2^ see Cubero et al. (2019) for a general argument underlying this statement. Therefore, one way to quantify the richness of the dynamic response of a neuron is to count the number of different dynamic states it undergoes in the course of the experiment. A proxy of this quantity is given by the variability of the spike frequency *k*_*s*_, that again can be measured in terms of an entropy

2 $$ H[K] = -\sum\limits_{k=1}^{M} \frac{k m_{k}}{M} \log_{M}\frac{k m_{k}}{M}.  $$

where *m*_*k*_ indicates the number of time bins containing *k* spikes,^3^ so that *km*_*k*_/*M* is the fraction of spikes that fall in bins with *k*_*s*_ = *k*. Again, rather than considering *H*[*K*] as a Shannon entropy of an underlying distribution *p*_*k*_ ≈ *km*_*k*_/*M* of spike frequencies, we take *H*[*K*] as an information theoretic measure of the information each spike contains on the dynamic state of the neuron at a given resolution.^4^Cubero et al. (2019) show that *H*[*K*] provides an upper bound to the number of informative bits that the data contains on the generative process. Also *H*[*K*] correlates with the number of parameters a model would require to describe properly the dataset, without overfitting (Haimovici and Marsili 2015). Hence, following Cubero et al. (2019), we shall call *H*[*s*] as *resolution* and *H*[*K*] as *relevance*.

In the current context, the reason for this choice can be understood as follows. In a given task or behavior, different neurons can have activities that are more or less related to the behavioral or neuronal states that are being probed in the experiment. Neurons that are *relevant* for encoding the animal’s behavior or task are expected to display rich dynamical responses, i.e. to have a large *H*[*K*]. On the contrary, neurons that are not involved in the animal’s behavior are expected to visit relatively fewer dynamic states, i.e. to have a lower *H*[*K*].

Notice that for very small binning times Δ*t* ≤Δ*t*_−_ (when each time bins contains at most one spike, i.e. *m*_*k*= 1_ = *M* and $m_{k^{\prime }}=0, ~\forall ~k^{\prime } > 1$) we find *H*[*K*] = 0 (and *H*[*s*] = 1). At the opposite extreme, when Δ*t* ≥Δ*t*_+_ and *H*[*s*] = 0, we have all spikes in the same bin, i.e. *m*_*k*_ = 0 for all *k* = 1,2,…,*M* − 1 and *m*_*M*_ = 1. Therefore again we find *H*[*K*] = 0. Hence, no information on the relevance of the neuron can be extracted at time scales smaller than Δ*t*_−_ or larger than Δ*t*_+_. At intermediate scales Δ*t* ∈ [Δ*t*_−_,Δ*t*_+_], *H*[*K*] takes non-zero values that we take as a measure of the relevance of the neuron for the freely-behaving animals being studied, at time scale Δ*t*.

**Fig. 1:**
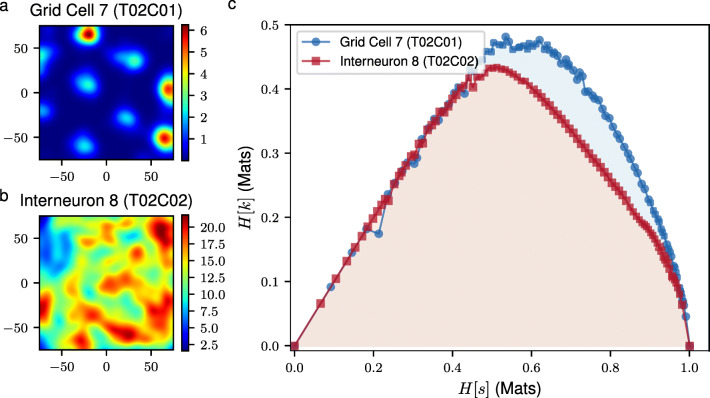
Proof of concept of the MSR as a relative information content measure. The smoothed firing rate maps of a grid cell (**a**) and an interneuron (**b**) in the mEC illustrates the spatial modulation of neural activity. Panel **c** shows the curves traced by the grid cell (blue) and interneuron (red). Each point, (*H*[*s*],*H*[*K*]), in this curve corresponds to a fixed binning time, Δ*t*, with which we see the corresponding temporal neural spike codes

Yet, the relevant time scale Δ*t* for a neuronal response to a stimulus may not be known *a priori* and/or the latter may evoke a dynamic response that spans multiple time scales. For this reason, we vary the binning time Δ*t* thereby inspecting multiple time scales with which we want to see the temporal code. As we vary Δ*t*, we can trace a curve in the *H*[*s*]-*H*[*K*] space for every neuron in the sample. Neurons with broad distributions of spike frequencies across different time scales will trace higher curves in this space and in turn, will cover larger areas under this curve (see Fig. 1c). Henceforth, we shall call the area under this curve as the *multiscale relevance* (MSR), $\mathcal {R}_{t}$. The *relevant neurons* (RNs), those with high values of $\mathcal {R}_{t}$, are expected to exhibit spiking behaviors that can be well-discriminated by downstream neural information processing units over short and long time scales and thus, are expected to be *relevant* to the encoding of higher representations. On the theoretical note, Marsili et al. (2013) show that, for a given value of *M* and of the resolution *H*[*s*], data that are maximally informative on the generative process are those for which *H*[*K*] takes a maximal value. In the high resolution region (small Δ*t* or large *H*[*s*]), the frequency distributions that achieve maximal values of *H*[*K*] are broad. More precisely, the frequency distribution behaves as $m_{k}\sim k^{-\mu -1}$ where − *μ* is the slope of the *H*[*K*] − *H*[*s*] curve. Indeed, *μ* quantifies the tradeoff between resolution (*H*[*s*]) and relevance (*H*[*K*]) in the sense that a reduction in *H*[*s*] of one bit delivers an increase of *μ* bits in *H*[*K*] (Cubero et al. 2019).

MSR is designed to capture non-trivial structures in the spike train stemming from the variations in spike rates. As such, it is expected to correlate with other measures characterizing temporal structure, such as bursty-ness and memory (Goh and Barabási 2008) and the coefficient of local variation in the interspike interval (Shinomoto et al. 2003, 2005) (see Supplementary Materials Text S1 for details). We have observed that, in synthetic data with given characteristics, MSR captures both the bursty-ness and memory of a time series, and local variations in the interspike intervals (see Supplementary Materials Fig. S1a,b and Supplementary Text S1 for definition). In addition, we find, in both synthetic and real data, a negative relation between MSR and spike frequency (i.e. *M*), which is partly associated with bursty-ness. Finally, we performed extensive tests on synthetically generated time series to show that the MSR captures non-trivial structure induced by the dependence of neural activity on external covariates (see Supplementary Materials Fig. S1g and the discussion on Fig. 6).

As a proof of concept of the MSR for featureless neural selection, we considered two neurons recorded simultaneously from the medial entorhinal cortex (mEC) by Stensola et al. (2012) – a grid cell (T02C01) and an interneuron (T02C02) – both of which were measured from the same tetrode and thus, are in close proximity in the brain region. The mEC and its nearby brain regions are notable for neurons that exhibit spatially selective firing (e.g., *grid cells* and *border cells*) which provides the brain with a locational representation of the organism and provides the hippocampus with its main cortical inputs. Grid cells have spatially selective firing behaviors that form a hexagonal pattern which spans the environment where the rat freely explores as in Fig. 1a. Apart from spatial information, grid cells can also be attuned to the HD especially in deeper layers of the mEC (Sargolini et al. 2006). These cells altogether provide the organism with an internal map which it then uses for navigation. On the other hand, interneurons, as in Fig. 1b, are inhibitory neurons which are still important towards the formation of grid cell patterns (Couey et al. 2013; Pastoll et al. 2013; Roudi and Moser 2014) but have much less spatially specific firing patterns. Intuitively, as the mEC functions as a hub for memory and navigation, grid cells, which provide the brain with a representation of space, should be more relevant for a downstream information processing “neuron” (possibly the place cells in the hippocampus) in encoding higher representations compared to interneurons. Indeed, the grid cell traces a higher curve in the *H*[*s*] − *H*[*K*] diagram of Fig. 1c, thus enclosing a larger area, as compared to the interneuron.

## Results

Following the observations in Fig. 1, we sought to characterize the temporal firing behavior of the 65 neurons which were simultaneously recorded from the mEC and its nearby regions of a freely-behaving rat as it explored a square area of length 150 cm (Stensola et al. 2012). This neural ensemble, as functionally categorized by Stensola et al. (2012), consisted of 23 grid cells, 5 interneurons, 1 putative border cell and 36 unclassified neurons, some of which had highly spatially tuned firing and nearly hexagonal spatial firing patterns (Stensola et al. 2012; Dunn et al. 2015, 2017). This dataset was chosen among the multiple recording sessions performed by Stensola et al. (2012) as this contained the most grid cells to be simultaneously recorded.

These results were then corroborated by characterizing the temporal firing behaviors of the 746 neurons which were recorded from multiple anterior thalamic nuclei areas, mainly the anterodorsal (AD) nucleus, and subicular areas, mainly the post-subiculum (PoS) of 6 different mice across 31 recording sessions while the mouse explored a rectangular area of dimensions 53 cm × 46 cm (Peyrache et al. 2015). This data was chosen as these heterogeneous neural ensembles contained a number of *HD cells* which are neurons that are highly attuned to HD.

Before showing the results on these data sets, we note that the the MSR is a robust measure. To establish this, we compared the MSRs computed using only the first half of the data to that computed from the second half. We obtained very similar results, confirming that the MSR is a reliable measure that can be used to score neurons (see Supplementary Materials Fig. S2a).

### MSR captures information on functionally relevant external correlates

As the mEC is crucial to spatial navigation, we sought to find whether the wide variations of neural firing as captured by the MSR would contribute towards a representation of the animal’s spatial organization, in one way or another. Different measures relating the spatial position, **x**, with neural activity had been employed in the literature to characterize spatially specific neural discharges, like the Skaggs-McNaughton spatial information, *I*(*s*,**x**) defined in Eq. (7) and by Skaggs et al. (1993), spatial sparsity measure, *sp*_**x**_ defined in Eq. (9) and by Skaggs et al. (1996) and Buetfering et al. (2014) and grid score, *g*, defined in Eq. (10) and by Sargolini et al. (2006), Dunn et al. (2017), Solstad et al. (2008) and Langston et al. (2010).

Apart from spatial location, HD also plays a crucial role in spatial navigation. The mean vector length, *R* (Eq. (11) in Section 5.4) is commonly used as a measure of HD selectivity of the activity of neurons. However, this measure assumes that there is only one preferred HD in which a given neuron is tuned to. Hence, we calculated two measures – the HD information, *I*(*s*,*𝜃*), and HD sparsity, *sp*_*𝜃*_ – inspired by the spatial information and spatial sparsity to quantify the information and selectivity of neural firing to HD respectively. These measures ought to detect non-trivial and multimodal HD tuning which may also be important in representing HD in the brain (Hardcastle et al. 2017).

**Fig. 2:**
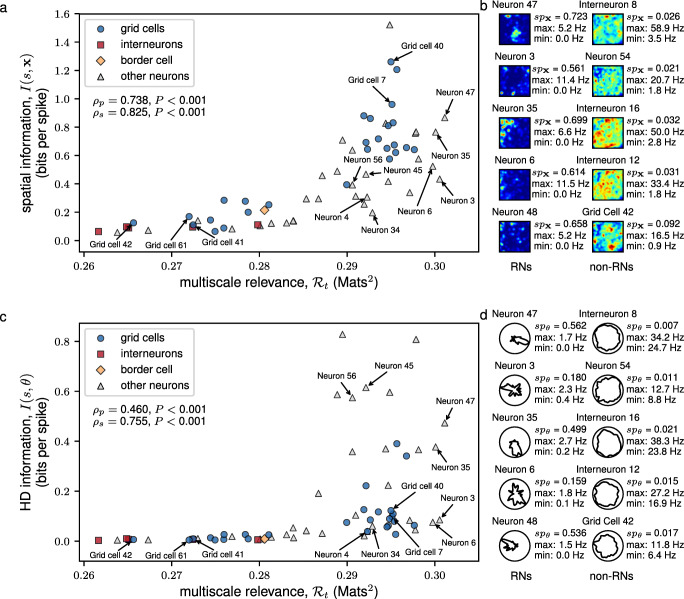
The MSR identified neurons that are spatially and head directionally informative. A scatter plot of the MSR vs. the spatial (HD) information is shown in **a** (**c**). The shapes of the scatter points indicate the identity of the neuron according to Stensola et al. (2012). The linearity and monotonicity of the multiscale relevance and the information measures were assessed by the Pearson’s correlation, *ρ*_*p*_, and the Spearman’s correlation, *ρ*_*s*_, respectively. Information bias was measured by a bootstrapping method, i.e., calculating the average of the spatial or head directional information of 1000 randomized spike trains. The spatial firing rate maps (HD tuning curves) of the 5 most relevant neurons (RNs) and the 5 most irrelevant neurons (non-RNs) are shown together in panel **b** (**d**) together with the calculated spatial sparsity, *sp*_**x**_, (HD sparsity, *sp*_*𝜃*_) and maximum and minimum firing

Figure 2 reports the spatial information (a) and the HD information (c) as a function of the MSR for each neuron in the mEC data. Figure 2b and d report the spatial firing rate maps and HD tuning curves for the top five RNs (left panel) and non-RNs (right panel) by MSR score, respectively (See also Supplementary Materials Figs. S4 and S5). We observed that non-RNs had very non-specific spatial and HD discharges as indicated by their sparsity scores (Fig. 2b and d, Supplementary Materials Figs. S4 and S5) whereas RNs had a broader range of spatial and HD sparsity (Fig. 2b and e, Supplementary Materials Figs. S4 and S5).

While we have observed that the MSR has a negative relation with the spike frequency (i.e. *M*), an analysis of the residual MSR revealed that the logarithm of the spike frequency (i.e., $\log M$) could not explain all of the variations in the MSR for the neurons in the mEC. We have seen that the residual MSRs (with respect to $\log M$) appeared to be correlated with spatial and HD information (see Supplementary Materials Figs. S2b-d).

Although local variations in the interspike intervals, as measured by *L*_*V*_, could still capture spatial and HD information (see Supplementary Materials Figs. S3a and b, respectively), we observed that the strength of correlation was stronger for MSR than for *L*_*V*_. While there is a positive correlation between *L*_*V*_ and the MSR (see Supplementary Materials Fig. S2e), we found that local variations could not explain what is captured by the MSR. In addition, the residual MSRs (with respect to *L*_*V*_) were observed to still be correlated with spatial or HD information (see Supplementary Materials Figs. S2f and g).

We found that (i) Neurons with high spatial information or high HD information also had high MSR, but the converse was not true. While there were highly RNs that responded exquisitely to space (grid cells 7 and 40) or HD (neurons 45 and 56) alone, the majority (e.g. neurons 35 and 47) encoded significantly both spatial and HD information. Secondly, we found that *ii)* Neurons with low MSR had both low spatial and low HD information (Fig. 2b and d, right panel), but again, the converse was not true (e.g. neurons 4 and 34). Finally *iii)* we found that some neurons, for example, neurons 3 and 6, despite having some spatial and HD sparsity as indicated in their rate maps (Fig. 2b and d, left panel), had relatively low spatial and HD information but were both identified to be RNs by MSR. This high MSR suggests that perhaps these neurons responded to other correlates involved in navigation different from spatial location or HD.

**Fig. 3:**
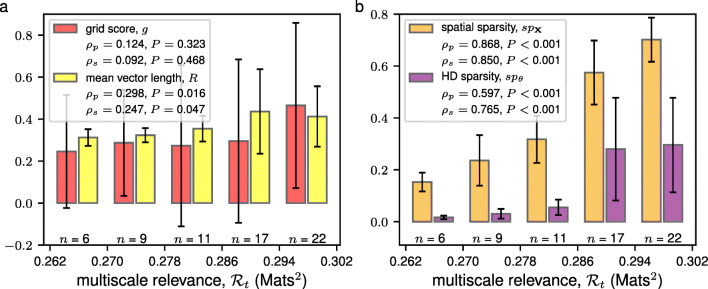
The MSR identified neurons with spatially and head directionally selective discharges. Bar plots depict the mean (height of the bar) along with the standard deviation (black error bars) of the grid score (red) and Rayleigh mean vector length (yellow) in panel **a**, and the spatial sparsity (orange) and HD sparsity (purple) in panel **b** for each neuron in the mEC within the relevance range as indicated. The relevance range was determined by equally dividing the range of the calculated MSR into 5 equal parts. The number of neurons whose MSRs fall within a relevance range is indicated below each bar. The linearity and monotonicity between the MSR and the different spatial and HD quantities were quantified using the Pearson’s correlation, *ρ*_*p*_, and the Spearman’s correlation, *ρ*_*s*_, respectively

Many of the grid cells were spotted as RNs, but not all. For example, grid cells 41, 42 and 61, that had a significant grid score, had a low MSR and a low spatial information. This indicated that different measures correlate differently with MSR. Figure 3 reports the distribution of the other four measures analyzed in this study conditional to different levels of MSR. Figure 3a shows that grid score maintains a large variation across all scales of the MSR, with a moderate increase in its average. A similar behavior was observed in Fig. 3a for the mean vector length. The converse is also true. For example, grid cells 33 and 41 have the same grid score but very different value of the MSR and of the spatial and HD information. A closer inspection of their rate maps (see Fig. S4) substantiates these differences.^5^ The *H*[*s*] − *H*[*K*] curve for neuron 33 stays above the one for neuron 41 at all values of *H*[*s*] (see Supplementary Materials Fig. S9a). High MSR neurons have very similar *H*[*s*] − *H*[*K*] curves, which saturate maximal achievable *H*[*K*] (Cubero et al. 2019), whereas low MSR neurons differ in characteristic ways. In particular, most of the interneurons feature the same linear *H*[*s*] − *H*[*K*] relation over an extended range of *H*[*s*] shown in Fig. 1c for interneuron 8 (see Supplementary Materials Fig. S9d).

Spatial sparsity and HD sparsity, instead, exhibit a significant correlation with the MSR as seen in Fig. 3b. The observation that RNs with highly sparse firing may have either low mean vector lengths or low grid scores was an indication that a non-trivial variabilities in firing behaviors does not necessarily obey the imposed symmetries of the tuning curves.

**Fig. 4:**
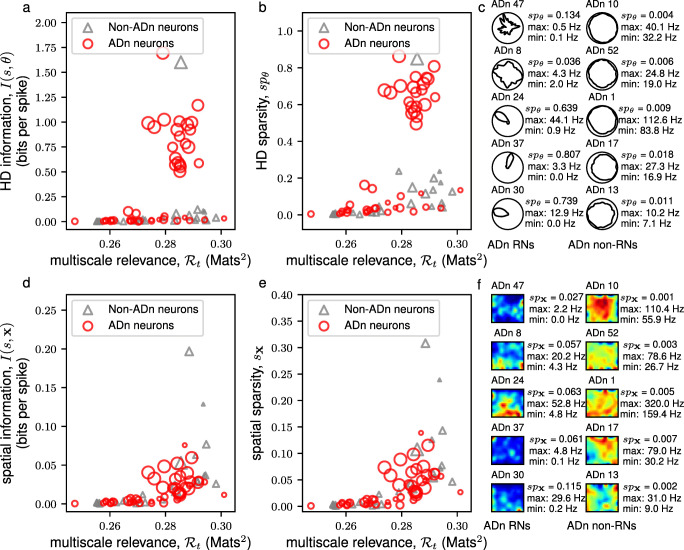
MSR of neurons from the anterodorsal thalamic nucleus (ADn) of Mouse 12 from a single recording session (Session 120806). A scatter plot of the multiscale relevance vs. the HD (spatial) information is shown in **a** (**d**). This plot is supplemented by a scatter plot between the MSR and HD (spatial) sparsity shown in **b** (**e**). The sizes of the scatter points reflect the mean vector length of the neural activity where the larger scatter points correspond to a sharp preferential firing to a single direction. The HD tuning curves (spatial firing rate maps) of the 5 most relevant neurons (RNs) and the 5 most irrelevant neurons (non-RNs) are shown together in panel **c** (**f**) together with the calculated HD sparsity, *sp*_*𝜃*_, (spatial sparsity, *sp*_**x**_) and maximum and minimum firing

**Fig. 5:**
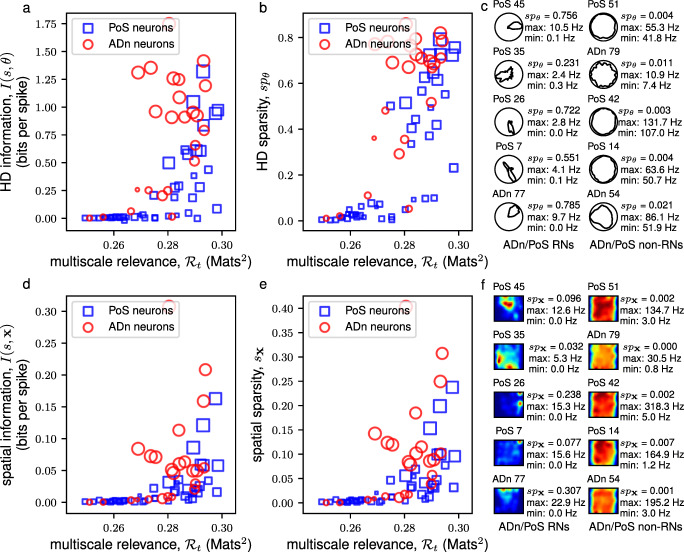
MSR of neurons from the anterodorsal thalamic nucleus (ADn) and post-subiculum (PoS) of Mouse 28 from a single recording session (Session 140313). A scatter plot of the MSR vs. the HD (spatial) information is shown in **a** (**d**). This plot is supplemented by a scatter plot between the MSR and HD (spatial) sparsity shown in **b** (**e**). The sizes of the scatter points reflect the mean vector length of the neural activity where the larger scatter points correspond to putative head direction cells while the shapes of the scatter points indicate the region where the neuron units were recorded from Peyrache et al. (2015) and Peyrache and Buzsáki (2015). The HD tuning curves (spatial firing rate maps) of the 5 most relevant neurons (RNs) and the 5 most irrelevant neurons (non-RNs) are shown together in panel **c** (**f**) together with the calculated HD sparsity, *sp*_*𝜃*_, (spatial sparsity, *sp*_**x**_) and maximum and minimum firing

Following the observations in the mEC, we turned to other regions in the brain – the thalamus – to check whether the non-trivial variability revealed by the MSR in the neural spiking, captured functionally relevant external correlates. To this, we analyzed the neurons in the ADn and PoS areas of freely behaving and navigating rodents. These regions are known to contain cells that robustly fire when the animal’s head is facing a specific direction (Taube et al. 1990; Taube 1995) and is believed to be crucial to the formation of grid cells in the mEC (Sargolini et al. 2006; Langston et al. 2010; McNaughton et al. 2006). Thus, we sought to find whether the variability as measured by the MSR contains signals of HD tuning. We observed that, in all of the 6 mice that were analyzed, the neurons having HD specific firing, i.e., neurons having high HD sparsity and high mean vector lengths, were RNs (see Supplementary Materials Fig. S6). Focusing on a subset of neurons of Mouse 12 (in Supplementary Materials Fig. S6a) that were simultaneously recorded in a single session (Session 120806), we observed, as in Fig. 4a,b, that HD attuned neurons were RNs. However, the HD alone may not explain the structure of the spike frequencies of these neurons (Peyrache et al. 2017). Hence, we also sought to find whether some of these neurons are spatially tuned. As seen in Fig. 4d,e, we found that some of the RNs were also modulated by the spatial location of the mouse. These results were also consistent for a subset of neurons of Mouse 28 (in Supplementary Materials Fig. S6f) that were simultaneously recorded in a single session (Session 140313) as in Fig. 5.

**Fig. 6:**
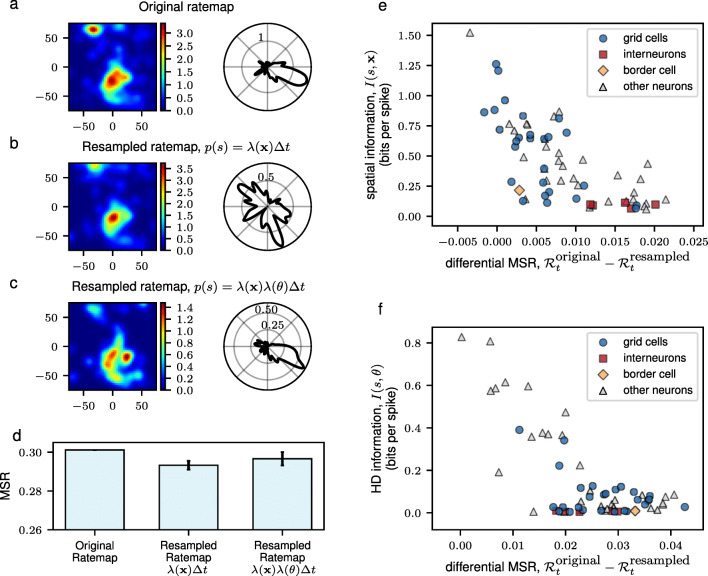
The MSR is a measure of information content of the neural activity. Resampling the firing rate map using spatial position only or in combination with HD resulted to a firing activity that closely resembled the actual firing pattern of mEC Neuron 47. Compared to the original firing rate maps in **a**, the spatial (left panels) and HD (right panels) firing rate maps were recovered by the resampling procedure in **b** and **c**. The result for a single realization of the resampling procedure is shown. (**d**) Bar plots show the MSR calculated from the original spiking activity of the neuron and the resampled rate maps. The mean and standard deviation of 100 realizations of the resampling procedure is reported. Scatter plot between the difference of the MSRs of the original spikes and of the synthetic spikes, resampled using only positional information (only HD information), for each neuron and the spatial information is shown in **e** (**f**)

To assess whether the variations in the spike frequencies, as characterized by the MSR, contained information about external stimuli relevant to navigation, we generated synthetic time series from idealized HD cells and found that neurons with a sharper HD tuning curves have both higher mutual information and higher MSR (see Supplementary Materials Fig. S1g). Following this observation, we resampled the spike count code of the neurons in the mEC such that only spatial information, or only HD information, or both spatial and HD information were incorporated. This resampling of the neural spiking was done by generating synthetic spikes assuming a non-homogeneous Poisson spiking with rates taken from the computed spatial firing rate maps and HD tuning curves (see Section 5.5). These assumptions were able to approximately recover the original rate maps as seen in Fig. 6b and c. Here, we focused our attention on *mEC Neuron 47* in the mEC data which had the highest MSR and also had both high spatial and high HD information. However, the same observation can be applied to other neurons that had both high spatial and HD information.

By resampling solely the spatial firing rate map as in Fig. 6d, we saw a decrease in the MSR despite having as much spatial information as the original code. When HD information was incorporated into the resampled spike frequencies, assuming the factorization of the firing probabilities due to position and HD, we observed an increase in the MSR for *Neuron 47*, almost up to the MSR for the original code. Such increase reveals the additional structure added onto the spiking activity of the resampled neuron. These findings support the idea that the temporal structure of the spike counts of the neuron, as measured by the MSR, come from its tuning profiles for both position and HD.

We also assessed which cells among the neurons in the mEC have MSRs that could be explained well by the spatial information and thus, were highly spatially attuned. We resampled the spatial firing rate maps of each of the neurons in the mEC data (see Section 5.5). The absolute difference between the original and resampled MSR, $ \mathcal {R}_{t}^{\text {original}} - \mathcal {R}_{t}^{\text {resampled}} $, was then computed from the resampled spikes. When the variations in the spike frequencies could be explained by the spatial firing fields, we expected this difference to be close to zero. As seen in Fig. 6e, we found that neurons having either high spatial (Fig. 6e) or HD (Fig. 6f) information tended to have a value of the differential MSR $ \mathcal {R}_{t}^{\text {original}} - \mathcal {R}_{t}^{\text {resampled}} $ close to zero. We also observed that most of the neurons having low differential MSRs were grid cells. The same observations could be drawn when resampling the HD tuning curves of each of the neurons in the mEC data. In particular, we also found that neurons having high HD information had differential MSRs close to zero as in Fig. 6f.

Taken altogether, these results suggest that the MSR can be used to identify the interesting neurons in a heterogeneous ensemble. The proposed measure is able to capture the non-trivial spike frequency distribution across multiple scales whose structure is highly influenced by external correlates that modulate the neural activity. Indeed, these analyses show that the MSR is able to capture information content of the neural spike code.

### Relevant neurons decode the external correlates as efficiently as informative neurons

We found in the previous section that neurons with low MSR had low spatial or HD information while higher MSR could indicate low or high values of spatial or HD information. In this section, we show that despite this, high MSR can still be used to select neurons that decode position or HD well. In other words, although high MSR can imply low spatial or HD information, in terms of population decoding, the highly RNs (selected based on only spike frequencies) performs equally well compared to the highly informative neurons (INs, selected using the knowledge of the external covariate).

To understand whether MSR could identify neurons in mEC whose firing activity allows the animal to identify its position, we compared the decoding efficiency of the 20 neurons with the highest MSR (top RNs) with that of the 20 neurons with the highest spatial information (top spatial INs) wherein the two sets overlap on 14 neurons (see Supplementary Materials Fig. S7a).

**Fig. 7:**
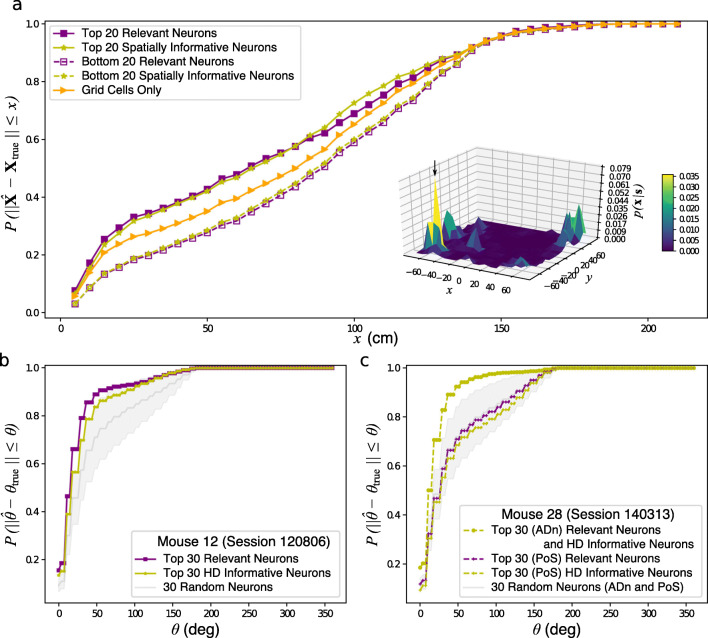
Positional decoding of RNs and INs in the mEC and HD decoding of the RNs and INs in the ADn of Mouse 12 and the ADn and PoS of Mouse 28 under a single recording session. Panel **a** shows the cumulative distribution of the decoding error, $\| \hat {{\mathbf X}} - {{\mathbf X}}_{true} \|$, for the RNs (solid violet squares) and spatially INs (solid yellow stars) neurons as well as for the non-RNs (dashed violet squares) and non-INs (dashed yellow stars). Spatial decoding was also performed for the 27 grid cells in the mEC data (solid orange triangles). The low positional decoding efficiency at some time points can be traced to the posterior distribution, *p*(**x**|**s**), of the rat’s position given the neural responses which exhibited multiple peaks as shown in the inset surface plot. For this particular example, the true position was found close to the maximal point of the surface plot as indicated by the arrows although such was not always the case. Panel **b** depicts the cumulative distribution of the decoding errors of the 30 RNs (violet squares) and 30 HD INs (yellow stars) in the ADn of Mouse 12 in Session 120806. The mean and standard errors of the cumulative distribution of decoding errors of 30 randomly selected ADn neuron (*n* = 1000 realizations) are shown in grey. On the other hand, panel **c** depicts the cumulative decoding error distribution of the 30 RNs (violet squares) and 30 HD INs in the ADn (yellow crosses) and PoS (yellow circles) of Mouse 28 in Session 140313. The mean and standard errors of the cumulative distribution of decoding errors of 30 randomly selected ADn or PoS neuron (*n* = 1000 realizations) are shown in grey. As the random selection included neurons from the ADn, which contain a pure head directional information and can decode the positions better than the neurons in the PoS, the decoding errors from the 30 randomly selected neurons were, on average, comparable to that of the relevant or head directionally informative PoS neurons. In all the decoding procedures, time points where all the neurons in the ensemble was silent were discarded in the decoding process

To this end, we employed a Bayesian approach to positional decoding wherein the estimated position at the *j*^th^ time bin, $\hat {{\mathbf x}}_{j}$, is determined by the position, **x**_*j*_, which maximizes an *a posteriori* distribution, *p*(**x**_*j*_|**s**_*j*_), conditioned on the spike pattern, **s**_*j*_, of a neural ensemble within the *j*^th^ time bin i.e.,

3 $$ \hat{{\mathbf x}}_{j} = \arg \max_{{{\mathbf x}}_{j}} p({{\mathbf x}}_{j} \vert {{\mathbf s}}_{j} ) = \arg \max_{{{\mathbf x}}_{j}} p({{\mathbf s}}_{j} \vert {{\mathbf x}}_{j} )p({{\mathbf x}}_{j})  $$

where the last term is due to Bayes rule, *p*(**s**_*j*_|**x**_*j*_) is the likelihood of a spike pattern, **s**_*j*_, given the position, **x**_*j*_, which depends on a given neuron model and *p*(**x**_*j*_) is the positional occupation probability which can be estimated directly from the data. Figure 7a shows that the top RNs decoded just as efficient as the top spatial INs. It can also be observed that the top RNs decode the positions better than the ensemble composed solely of grid cells.

Because of the sizable overlap between the top RNs and the top spatial INs, one might argue that much of the spatial information needed for positional decoding is concentrated on the neurons in the overlap (ONs). To address this, we randomly selected 6 neurons among the mEC neurons outside the overlap and, together with the 14 ONs, decoded for the position as done above. If the positional decoding information is contained in the ONs, then we should observe the same decoding efficiency as either the top RNs or top spatial INs. However, we found that the decoding efficiency of the ONs decreased (see Supplementary Materials Fig. S7d). We also found that for the decoded positions within 5 cm from the true position, the decoding efficiency of the top RNs were up to 4*σ* from the mean decoding efficiency of the ONs, as measured by the *z*-score compared to that of the top spatial INs which was at around 2*σ*. This indicates that the 6 RNs outside the overlap provide better decodable spatial representation than those of the 6 spatial INs.

Because local variations in the interspike intervals of the neurons in the mEC correlated with spatial information, we sought to find whether neurons with high local variations (LVNs) also contained decodable spatial representation. We took the top 20 LVNs in the mEC and decoded for the position as done above. We found that the decoding efficiency of top LVNs are much lower compared to top RNs (see Supplementary Materials Fig. S3c). This indicates that the repertoire of responses coming from local variations in the interspike intervals of mEC neurons alone can not represent space in freely-behaving rats.

To substantiate the decoding results obtained for neurons in the mEC, we also took the ADn RNs and the HD INs in the of Mouse 12 (Session 120806) in Fig. 4 to decode for HD. Mouse 12 was chosen as this animal had the most HD cells recorded among the mice that only had recordings in the ADn (Peyrache and Buzsáki 2015). In particular, we looked at the HD decoding at longer time scales (in this case, Δ*t* = 100 ms), where we could model the neural activity using a Poisson distribution, *p*(**n**_*j*_|*𝜃*_*j*_) similar to that in Eq. (15). Bayesian decoding adopts an equation

4 $$ \hat{\theta}_{j} = \arg \max_{\theta_{j}} p(\theta_{j} \vert {{\mathbf n}}_{j} ) = \arg \max_{\theta_{j}} p({{\mathbf n}}_{j} \vert \theta_{j} )p(\theta_{j}) $$

similar to Eq. (3) to estimate the decoded HD, $\hat {\theta }_{j}$, where *p*(*𝜃*_*j*_) is the HD occupation as estimated from the data. We compared the decoding efficiency of the 30 RNs with the 30 HD INs which had 22 neurons that are relevant (see Supplementary Materials Fig. S3b). We also compared the decoding efficiencies of the ADn RNs or HD INs with 30 randomly selected ADn neurons (*n* = 1000 realizations). As seen in Fig. 7b, the RNs decoded just as well as the neural population composed of HD INs. Furthermore, the decoding efficiency of the RNs were observed to be far better than the decoding efficiency of a random selection of neurons in the ensemble.

We also compared the decoding efficiency of the ADn and PoS neurons from Mouse 28 (Session 140313) which had the most HD cells recorded among the mice that had recordings in both ADn and PoS (Peyrache and Buzsáki 2015) as in Fig. 5. As seen in Fig. 7c, neurons in the ADn decoded the HD more efficiently than the neurons in the PoS. These results are consistent with the notion that the ADn contains pure HD modulation which allow for neurons in the ADn to better predict the mouse’s HD compared to the neurons in the PoS which contain, instead, true spatial information (Peyrache et al. 2015; Peyrache et al. 2017). For the neurons in Mouse 28 (Session 140313), it had to be noted that the 30 ADn RNs also happened to be the 30 ADn HD INs (see Supplementary Materials Fig. S7c). On the other hand, among the 30 PoS RNs, 23 were HD INs (also see Supplementary Materials Fig. S7c). We observed that the PoS RNs decode just as efficient as the PoS HD INs consistent with the findings for Mouse 12 (Session 120806).

Taken altogether, despite being blind to the rat’s position and of the mouse’s HD, the MSR is able to capture neurons that can decode the position and HD just as well as the spatial INs and as the HD INs.

## Discussion

In the present work, we introduced a novel, parameter-free and fully featureless method – which we called multiscale relevance (MSR) – to characterize the temporal structure of the activities of neurons within a heterogeneous population. We have shown that the neurons showing persistently broad spike frequency distributions across a wide range of time scales, as measured by the MSR, typically carry information about the external correlates related to the behavior of the observed animal. By analyzing the neurons in the mEC and nearby brain regions and the neurons in the ADn and PoS – areas in the brain that are pertinent to spatial navigation – we showed that the RNs in these regions have firing behaviors that are selective for spatial location and HD. Here, we found that in many cases, the neurons that display broad spike distributions tend to have conjugated representations in that they exhibit high mutual information with multiple behavioral features. These findings are consistent with those observed experimentally by Sargolini et al. (2006) and statistically by Hardcastle et al. (2017).

The fact that the MSR can be used to select informative neurons as well as neurons that show high decoding performance is consistent with the expectation that the information carried by the activity of a given neuron is encapsulated in the sole spike activity – the only information available to downstream neurons – to decode a representation of the feature space. This suggests that relevant neurons should feature a rich variety of long-ranged statistical patterns of the spike activity. This, in turn, results in broad frequency distributions at different time-scales, which are quantified by the relevance *H*[*K*], as discussed in Cubero et al. (2019). Hence, at a given resolution, as defined in (1), we estimate the complexity of the temporal code by the relevance defined in (2). Since natural and dynamic stimuli and behaviors often operate on multiple time scales, the MSR integrates over different resolution scales, thus allowing us to spot neurons exhibiting persistent non-trivial spike codes across a broad range of time scales.

Broad distributions of spike frequencies, characterized by a high MSR, exhibit a stochastic variablility that requires richer parametric models (Haimovici and Marsili 2015). In a decoding perspective, these non-trivial distributions afford a higher degree of distinguishability of neural responses to a given stimuli or behavior. Indeed, by decoding either spatial position or HD using statistical approaches, we found that the responses of the RNs allow downstream processing units to efficiently decode the external correlates just as well as the neurons whose resulting tuning maps contain information about those external correlates.

Finally, we observed that the population of relevant neurons, as identified by the MSR, is not homogeneous, e.g., the relevant neurons in the mEC data are not composed solely by grid cells and the relevant neurons in the ADn and PoS are not necessarily composed solely of HD cells. Noteworthy, the decoding efficiency of the relevant neurons was observed to be better compared to the ensemble comprising solely the grid cells. When taken altogether, these observations support the idea that population heterogeneity may play a role towards efficient encoding of stimuli (Chelaru and Dragoi 2008; Meshulam et al. 2017).

The fact that the MSR captures functional information from the temporal code is a remarkable aspect of this measure. This method can then be used as a pre-processing tool to impose a less stringent criteria compared to those widely used in many studies (e.g., mean vector length, spatial sparsity and grid scores) thereby directing further investigation to interesting neurons. The MSR is expected to be particularly useful in detecting relevant neurons in high-throughput studies – where the activity of many neurons are measured simultaneously or in single-electrode neural recordings where, under a given task, an experiment is done multiple times – where the function of neurons or the correlates they encode are not known *a priori*. Our discussion has been confined to correlates related to navigation, but it applies in a straightforward manner to other correlates (e.g. heart rate or pupil diameter), that may be responsible for the recorded activity of relevant neurons.

Whether this measure can also be used to identify functionally relevant neuronal units recorded through calcium imaging or through fMRI is also an exciting direction for future studies. A further promising direction lies in the extension of the principles used to construct MSR to the study of neural assemblies (Russo and Durstewitz 2017). This could allow one to probe the importance of correlated firing of neurons in representing external stimuli or behaviors. For example, the analysis of boolean functions of pairs of neurons in the mEC recordings, shown in the Supplementary Material (Fig. S8), suggests that the representations of individual neurons are non-redundant and that interneurons play a peculiar role in the information aggregation.

## Materials and methods

### Data collection

The data used in this study are recordings from rodents with multisite tetrode implants. These neurons are of particular interest because they are involved in spatial navigation.

#### Data from medial entorhinal cortex (mEC)

The spike times of 65 neurons recorded across the mEC area of a male Long Evans rat (Rat 14147) were taken from Stensola et al. (2012). The rat was allowed to freely explore a box of dimension 150 × 150 cm^2^ for a duration of around 20 mins. The positions were tracked using a platform attached to the head with red and green diodes fixed at both ends. Additional details about the data acquisition can be found in the paper by Stensola et al. (2012).

#### Data from the anterodorsal thalamic nucleus (ADn) and post-subiculum (PoS)

The spike times of 746 neurons recorded from multiple areas in the ADn and PoS across multiple sessions in six free moving mice (Mouse 12, Mouse 17, Mouse 20, Mouse 24, Mouse 25 and Mouse 28) while they freely foraged for food across an open environment with dimensions 53 × 46 cm^2^ and in their home cages during sleep were taken from Peyrache and Buzsáki (2015). Mouse 12, Mouse 17 and Mouse 20 only had recordings in the ADn while Mouse 24, Mouse 25 and Mouse 28 had simultaneous recordings from ADn and PoS. The positions were tracked using a platform attached to the heads of the mice with red and blue diodes fixed at both ends. Only the recorded spike times during awake sessions and the neural units with at least 100 observed spikes were considered in this study. Additional information regarding the data acquisition can be found in the paper by Peyrache et al. (2015) and the CRCNS^6^ database entry by Peyrache and Buzsáki (2015).

### Position and speed filtering

The position time series for the mEC data were smoothed to reduce jitter using a low-pass Hann window FIR filter with cutoff frequency of 2.0 Hz and kernel support of 13 taps (approximately 0.5 s) and were then renormalized to fill missing bins within the kernel duration as done by Dunn et al. (2017). The rat’s position was taken to be the average of the recorded and filtered positions of the two tracked diodes. The head direction was calculated as the angle of the perpendicular bisector of the line connecting the two diodes using the filtered positions. The speed at each time point was computed by dividing the trajectory length with the elapsed time within a 13-time point window. When calculating for spatial firing rate maps and spatial information (see below), only time points where the rat was running faster than 5 cm/s were considered. No speed filters were imposed when calculating for head directional tuning curves and head directional information. On the other hand, no position smoothing nor speed filtering were performed when calculating for the spatial firing rate maps and spatial information for the ADn and PoS data.

### Rate maps

The spike location, $\mathbf {\xi }_{j}^{(i)}$, of neuron *i* at a spike time $t_{j}^{(i)}$ was calculated by linearly interpolating the filtered position time series at the spike time. As done by Dunn et al. (2017), the spatial firing rate map at position **x** = (*x*,*y*) was calculated as the ratio of the kernel density estimates of the spatial spike frequency and the spatial occupancy, both binned using 3 cm square bins, as

5 $$ f({\mathbf x}) = \frac{{\sum}_{j=1}^{M} K({\mathbf x} \vert \mathbf{\xi}_{j} )}{{\sum}_{j=1}^{M} {\Delta} t_{j} K({{\mathbf x}} \vert {{\mathbf x}}_{j} )} $$

where a triweight kernel

6 $$ K({\mathbf x} \vert \mathbf{\xi} ) = \frac{4}{9{\pi\sigma_{K}^{2}}} \left[ 1 - \frac{ \| {\mathbf x} - \mathbf{\xi} \|^{2} }{9{\sigma_{K}^{2}}}\right]^{3}, \| {\mathbf x} - \mathbf{\xi} \| < 3\sigma_{K} $$

with bandwidth *σ*_*K*_ = 4.2 cm was used. In place of a triweight kernel, a Gaussian smoothing kernel with *σ*_*G*_ = 4.0 truncated at 4*σ*_*G*_ was also used to estimate the rate maps which gave qualitatively similar results. For better visualization, a Gaussian smoothing kernel with *σ*_*G*_ = 8.0 was used to filter the spatial firing rate map.

On the other hand, for head direction tuning curves, the angles were binned using 9^∘^ bins. The tuning curve was then calculated as the ratio of the head direction spike frequency and the head direction occupancy without any smoothing kernels as the head direction bins are sampled well-enough. For better visualization, a Gaussian kernel with smoothing window of 20^∘^ was used to filter the tuning curves.

### Information, sparsity and other scores

Given a feature, *ϕ* (e.g., spatial position, **x**, head direction, *𝜃* or speed, *v*), the information between the neural spiking **s** and the feature can be calculated á la Skaggs-McNaughton (Skaggs et al. 1993). In particular, under the assumption of a non-homogeneous Poisson process with feature dependent rates, *λ*(*ϕ*), under small time intervals Δ*t*, the amount of information, in bits per second, that can be decoded from the rate maps is given by

7 $$ I(s, \phi) = \sum\limits_{\phi} p(\phi)\frac{\lambda(\phi)}{\bar{\lambda}} \log{\frac{\lambda(\phi)}{\bar{\lambda}}}  $$

where *λ*(*ϕ*) is the firing rate at *ϕ*, *p*(*ϕ*) is the probability of occupying *ϕ* and

8 $$ \bar{\lambda} \equiv \sum\limits_{\phi} \lambda(\phi) p(\phi)  $$

is the average firing rate. To account for the bias due to finite samples, the information of a randomized spike frequency was calculated using a bootstrapping procedure. To this end, the spikes were randomly shuffled 1000 times and the information for each reshuffling was calculated. The average randomized information was then subtracted from the non-randomized information. It is interesting to mention, in passing, that reshuffling wipes all information between all correlates and the time of spiking. Since the MSR only depends on the timing of the spikes, and not on other correlates, is it unaffected by reshuffling.

Apart from the information, one of the measures that are used to quantify selectivity of neural firing to a given feature is the firing sparsity (Buetfering et al. 2014) which can be calculated using

9 $$ sp_{\phi} = 1 - \frac{ \left( {\sum}_{\phi} \lambda(\phi) p(\phi) \right)^{2}}{{\sum}_{\phi} \lambda(\phi)^{2} p(\phi)}.  $$

Apart from the measures of information and sparsity, we also calculated the grid scores, *g*, for the neurons in the mEC data. The grid score is designed to quantify the hexagonality of the spatial firing rate maps through the spatial autocorrelation maps (or autocorrelograms) and was first used by Sargolini et al. (2006) to identify putative grid cells. In brief, the grid score is computed from the spatial autocorrelogram where each element *ρ*_*ij*_ is the Pearson’s correlation of overlapping regions between the spatial firing rate map shifted *i* bins in the horizontal axis and *j* bins in the vertical axis and the unshifted rate map. The angular Pearson autocorrelation, acorr(*u*), of the spatial autocorrelogram was then calculated using spatial bins within a radius *u* from the center at lags (or rotations) of 30^∘^, 60^∘^, 90^∘^, 120^∘^ and 150^∘^, as well as the ± 3^∘^ and ± 6^∘^ offsets from these angles to account for sheared grid fields (Stensola et al. 2015). As done by Dunn et al. (2017), the grid score, *g*(*u*), for a fixed radius of *u*, is computed as

10 $$ \begin{array}{@{}rcl@{}} g(u) &=& \frac{1}{2} \left[ \max \lbrace \text{acorr}(u) \mathrm{~at~} 60^{\circ} \pm (0^{\circ}, 3^{\circ}, 6^{\circ}) \right. \\ &&\left. +\max \lbrace \text{acorr}(u) \mathrm{~at~} 120^{\circ} \pm (0^{\circ}, 3^{\circ}, 6^{\circ}) \right] \\ &&- \frac{1}{3} \left[ \min \lbrace \text{acorr}(u) \mathrm{~at~} 30^{\circ} \pm (0^{\circ}, 3^{\circ}, 6^{\circ}) \right. \\ &&\left. +\min \lbrace \text{acorr}(u) \mathrm{~at~} 90^{\circ} \pm (0^{\circ}, 3^{\circ}, 6^{\circ}) \right. \\ && \left. +\min \lbrace \text{acorr}(u) \mathrm{~at~} 150^{\circ} \pm (0^{\circ}, 3^{\circ}, 6^{\circ}) \right]. \end{array} $$

The final grid score, *g*, is then taken as the maximal grid score, *g*(*u*), within the interval *u* ∈ [12 cm,75 cm] in intervals of 3 cm.

Another quantity that was calculated in this paper is the Rayleigh mean vector length, *R*. Given the angles {*𝜃*_1_,…,*𝜃*_*M*_} where a neuronal spike was recorded, the mean vector length can be calculated as

11 $$ R = \sqrt{ \left( \frac{1}{M} \sum\limits_{i=1}^{M} \cos\theta_{i} \right)^{2} + \left( \frac{1}{M} \sum\limits_{i=1}^{M} \sin\theta_{i} \right)^{2} }.  $$

Note that for head direction cells where the neuron fires at a specific head direction, the angles will be mostly concentrated along the preferred head direction, *𝜃*_*c*_, and hence, *R* ≈ 1 whereas for neurons with no preferred direction, *R* ≈ 0.

### Resampling the firing rate map

The calculated rate maps and the real animal trajectory were used to resample the neural activity assuming non-homogeneous Poisson spiking statistics with rates taken from the rate maps. To this end, the real trajectory of the rat was divided into Δ*t* = 1 ms bins. The position and head direction were linearly interpolated from the filtered positions described above. The target firing rate, *f*_*j*_ in bin *j* was then calculated by evaluating the tuning profile at the interpolated position or head direction. Whenever the target firing rate was modulated by both the position and head direction, we assumed that the contribution due to each feature was multiplicative and thus, *f*_*j*_ is calculated as the product of the tuning profiles at the interpolated position and the interpolated head direction. A Bernoulli trial was then performed in each bin with a success probability given by *f*_*j*_Δ*t*.

### Statistical decoding

For positional decoding, we divided the space in a grid of 20 × 20 cells of 7.5 cm × 7.5 cm spatial resolution, which was comparable to the rat’s body length. Time was also discretized into 20 ms bins which ensured that for most of the time (i.e. in 92*%* of the cases), the rat was located within a single spatial cell. Under these time scales, the responses of a neuron can be regarded as being drawn from a binomial distribution, i.e., either the neuron *i* is active ($s_{j}^{(i)}=1$) or not ($s_{j}^{(i)}=0$) between (*j* − 1)Δ*t* and *j*Δ*t*. The likelihood of the neural responses, $\text {\textbf {s}}_{j} = (s_{j}^{(1)}, \ldots , s_{j}^{(N)})$ of *N* independent neurons at a given time conditioned on the position, **x**_*j*_ is then given by

12 $$ p({{\mathbf s}}_{j} \vert {{\mathbf x}}_{j} ) = \prod\limits_{i=1}^{N} (\lambda^{(i)}({{\mathbf x}}_{j}){\Delta} t)^{s^{(i)}_{j}} (1-\lambda^{(i)}({{\mathbf x}}_{j}){\Delta} t)^{1-s^{(i)}_{j}}  $$

where $\lambda ^{(i)}({{\mathbf x}}_{j})$ is the firing rate of neuron *i* at **x**_*j*_ estimated from its corresponding spatial firing rate map. Given the prior distribution on the position, *p*(**x**_*j*_), which is estimated from the data, the posterior distribution of the position, **x**_*j*_, given the neural responses, **s**_*j*_ at time *t* is given by

13 $$ p({{\mathbf x}}_{j} \vert {{\mathbf s}}_{j} ) = \frac{p({{\mathbf s}}_{j} \vert {{\mathbf x}}_{j} )p({{\mathbf x}}_{j})}{p({{\mathbf s}}_{j})}. $$

The decoded position, as in the Bayesian 1-step decoding by Zhang et al. (1998), was calculated as

14 $$ \hat{{\mathbf x}}_{j} = \arg \max_{{{\mathbf x}}_{j}} p({{\mathbf s}}_{j} \vert {{\mathbf x}}_{j} )p({{\mathbf x}}_{j}). $$

For head directional decoding, on the other hand, we divided the angles, *𝜃* ∈ [0,2*π*) in 9^∘^ bins. For this case, time was instead discretized into 100 ms bins. Under these time scales, the neurons could not be regarded simply as either active or not. Hence, it was natural to switch towards the analysis of population vectors, **n**_*j*_, a vector which represents the number of spikes, $n^{(i)}_{j}$, recorded from each neuron within the *j*^th^ time bin, to decode for the head direction. In this case, the number of spikes, $n^{(i)}_{j}$, that neuron *i* discharges between (*j* − 1)Δ*t* and *j*Δ*t* can be modeled as a non-homogeneous Poisson distribution

15 $$ p(n^{(i)}_{j} \vert \theta_{j} ) = \frac{\lambda^{(i)}(\theta_{j})^{n^{(i)}_{j}}}{n^{(i)}_{j}!} \exp(-\lambda^{(i)}(\theta_{j}))  $$

with *λ*^(*i*)^(*𝜃*_*j*_) being the firing rate of neuron *i* at *𝜃*_*j*_ estimated from the HD tuning curve, and thus, under the independent neuron assumption, $p({{\mathbf n}}_{j} \vert \theta _{j} ) = {\prod }_{i=1}^{N} p(n^{(i)}_{j} \vert \theta _{j} )$. The decoded head direction can then be calculated as

16 $$ \hat{\theta}_{j} = \arg \max_{\theta_{j}} p({{\mathbf n}}_{j} \vert \theta_{j} )p(\theta_{j}). $$

where *p*(*𝜃*_*j*_) is the head directional prior distribution which is estimated from the data. Note that in all of the decoding procedures, we only decoded for time points with which at least one neuron was active. Furthermore, the decoding exercise for both space and HD were done on different time bins, spanning from 10 ms to 200 ms, and obtained qualitatively similar results.

### Boolean function

Spikes from mEC neurons were binned and binarized at Δ*t* = 1ms time intervals such that each bin *B*_*s*_ takes a value of 1 if there is at least one recorded spike within the time interval [(*s* − 1)Δ*t*,*s*Δ*t*) and 0 otherwise. Given two neuron pairs *i* and *j*, a new spike train was built using the Boolean function rules in Table 1 to each time bin. The corresponding MSR, and spatial and HD information were then calculated. The same procedure was done using Δ*t* = 0.5 ms time intervals which gave qualitatively similar results. Note that the events where two neurons fire together (that are those identified by the AND function) are generally very sparse and do not allow for a reliable calculation of the MSR and thus, the MSR for pairs of neurons having at least 100 co-firing events were considered.

**Table 1:** Boolean function rules

*i*	*j*	AND(*i*,*j*)	OR(*i*,*j*)	XOR(*i*,*j*)
0	0	0	0	0
0	1	0	1	1
1	0	0	1	1
1	1	1	1	0

### Noise correlations

Spatial noise correlations were calculated as done by Dunn et al. (2015). In brief, spikes were binned at Δ*t* = 1ms time intervals and were smoothened by a Gaussian kernel with width of 20ms. The spatial environment was binned into a grid of 7.5 cm square tiles and the trajectories over the spatial bin *α*, defined as the time that the rat enters and leaves the square tile *α*, were noted. For each neuron *i*, a 1 × *k* vector, ${{\mathbf r}}_{i}^{\alpha }$, of the mean firing rate over each of the *k* trajectories was constructed. The spatial noise correlation between neuron pairs *i* and *j* were then calculated as

17 $$ C_{ij}({\mathbf x}) = \langle \rho_{P}({{\mathbf r}}_{i}^{\alpha}, {{\mathbf r}}_{j}^{\alpha}) \rangle_{\alpha} $$

where *ρ*_*P*_(**x**,**y**) is the Pearson’s correlation and the averages are taken over the spatial bins *α*.

### Source codes

All the calculations in this manuscript were done using personalized scripts written in Python 3. The source codes for calculating multiscale relevance (which is also compatible with Python 2) and for reproducing the figures in the main text are accessible online.^7^

### Electronic supplementary material

Below is the link to the electronic supplementary material.

(PDF 1.85 MB)

## Footnotes

1 With no prior knowledge, a spike can be any of the *M* possible spikes, so its *a priori* uncertainty is of $\log _{2} M$ bits. The information on which bin *s* the spike occurs, reduces the number of choices from *M* to *k*_*s*_ and the uncertainty to $\log _{2} k_{s}$ bits. Averaging the information gain $\log M-\log k_{s}$ over the *a priori* distribution of spikes and dividing by $\log M$, yields Eq. (1). It is also worth to stress that Eq. (1) does not refer to the estimate of the entropy of a hypothetical underlying distribution *p*_*s*_ from which spikes are drawn. This would not make much sense, because it is well-known that the *naïve* estimate of the Shannon entropy in Eq. (1) obtained with the maximum likelihood estimator $\hat p_{s}=k_{s}/M$ suffers from strong biases (Treves and Panzeri 1995; Strong et al. 1998).

2 One may argue that, if the activity in the previous bins *s* − 1 and $s^{\prime }-1$ differs considerably, then the dynamic state in bin *s* and $s^{\prime }$ may also be considered different. We take the view that this distinction is automatically taken into account when considering larger bins (i.e. Δ*t* → 2Δ*t*).

3 *m*_*k*_ satisfies the obvious relation ${\sum }_{k=0}^{M} k m_{k} = {\sum }_{s=1}^{T} k_{s} = M$.

4 Again, the knowledge of the associated dynamical state, i.e. the spike frequency *k* of the bin it belongs to, provides information to identify the timing of a spike by reducing the number of possible choices from *M* to *k**m*_*k*_, which is the number of spikes in bins with the same dynamical state *k*. The information gain is given by *H*[*K*].

5 Grid cell 41 has a higher firing rate, but also the residual MSR wrt to $\log M$ is significantly negative.

6 https://crcns.org

7 https://github.com/rcubero/MSR
