# Supplementary material for: Multiscale relevance and informative encoding in neuronal spike trains
Source: J Comput Neurosci. 2020 Jan 28;48(1):85–102. doi: 10.1007/s10827-020-00740-x (PMC7035307; doi:10.1007/s10827-020-00740-x)
Supplement: Supplementary file 1 — (PDF 1.85 MB) [file 10827_2020_740_MOESM1_ESM.pdf]

Supplementary Materials to *Multiscale relevance and informative encoding in populations of neurons*

Ryan John Cubero*[1,2,3,4], Matteo Marsili[2], and Yasser Roudi[1]

[1] Kavli Institute for Systems Neuroscience and Centre for Neural Computation, Norwegian University of Science and Technology (NTNU), Trondheim, Norway
[2] The Abdus Salam International Center for Theoretical Physics, Trieste, Italy
[3] Scuola Internazionale Superiore di Studi Avanzati, Trieste, Italy
[4] Present address: IST Austria, Klosterneuburg, Austria

*Corresponding author: ryanjohn.cubero@ist.ac.at

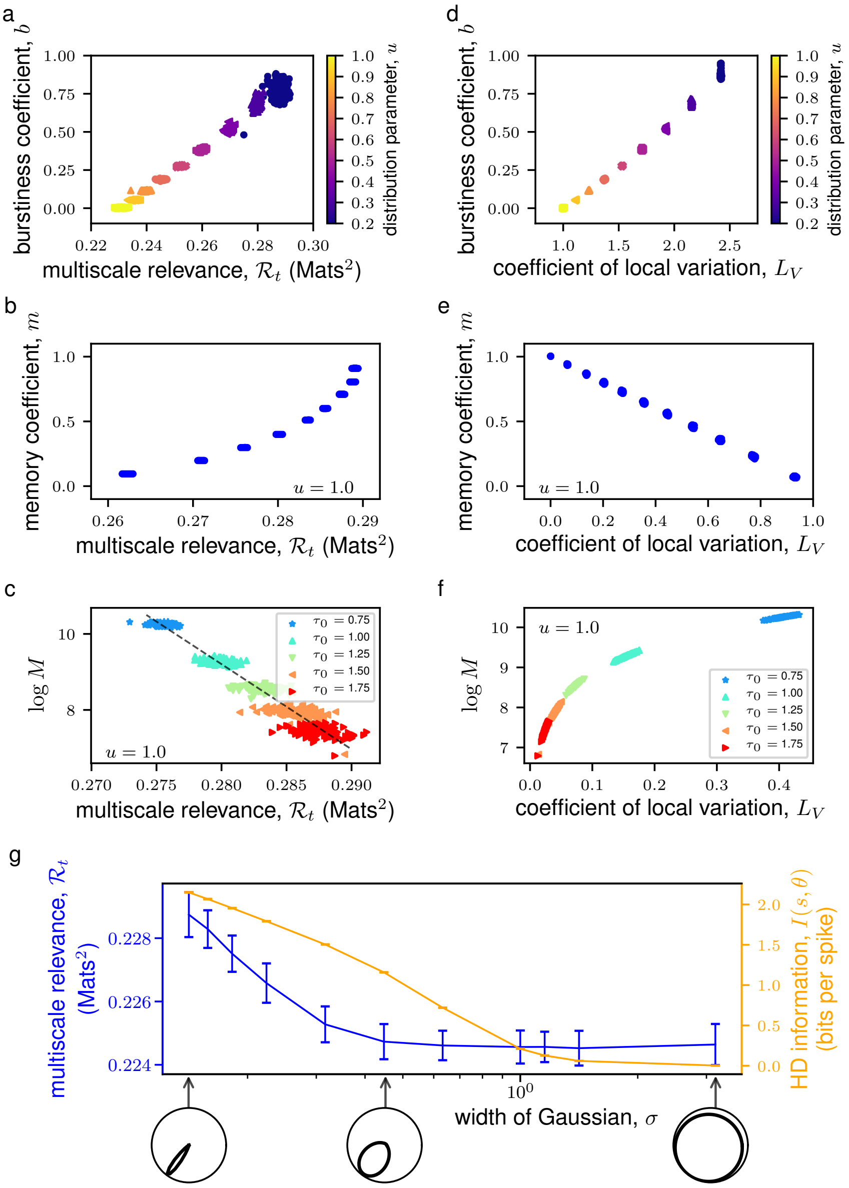

FIGURE S1. **Synthetically-generated neural data reveals relationship of the MSR and of the coefficient of local variation, L_V, with the bursty-ness and memory coefficients, and Skaggs-McNaughton mutual information.** Interevent times were drawn from a stretched exponential distribution to simulate random events up to 100,000 time units where short-term memory effects were introduced through a shuffling procedure and the number of random events, M, were varied by modifying the characteristic time constant, τ_0 (See Supplementary Text S1 for details). Scatter plots show how the multiscale relevance (MSR) scales with the bursty-ness coefficient, b (panel **a**), the memory coefficient, m (panel **b**), and $\log M$ (panel **c**). In panel **b**, random events were drawn from a stretched exponential distribution with $u = 1.0$ while in panel **c**, the parameter u was set to 0.3. Panels **d**, **e** and **f**, on the other hand, show the relationship between L_V and bursty-ness coefficient, memory coefficient and $\log M$ respectively. The results for 100 realizations of such random events are shown. Notice, in **c** and **f**, that both the MSR and the L_V are sensitive to the number of spiking events. Idealized HD cells were also simulated by assuming that the firing probability conditioned on the HD follows a circular Gaussian distribution centered at a preferred HD, θ_0, with a given width, σ. Spike train data was generated using resampling methods (See Section 5.5) using a random walk trajectory for a fixed θ_0 and σ. For each realization of the spike train data, the MSR (blue line) and the HD information (orange line) were calculated and shown in panel **g**. Here, we report the mean and standard deviation across 100 spike train realization. For a given θ_0, sample HD tuning curves are shown in the polar plots.

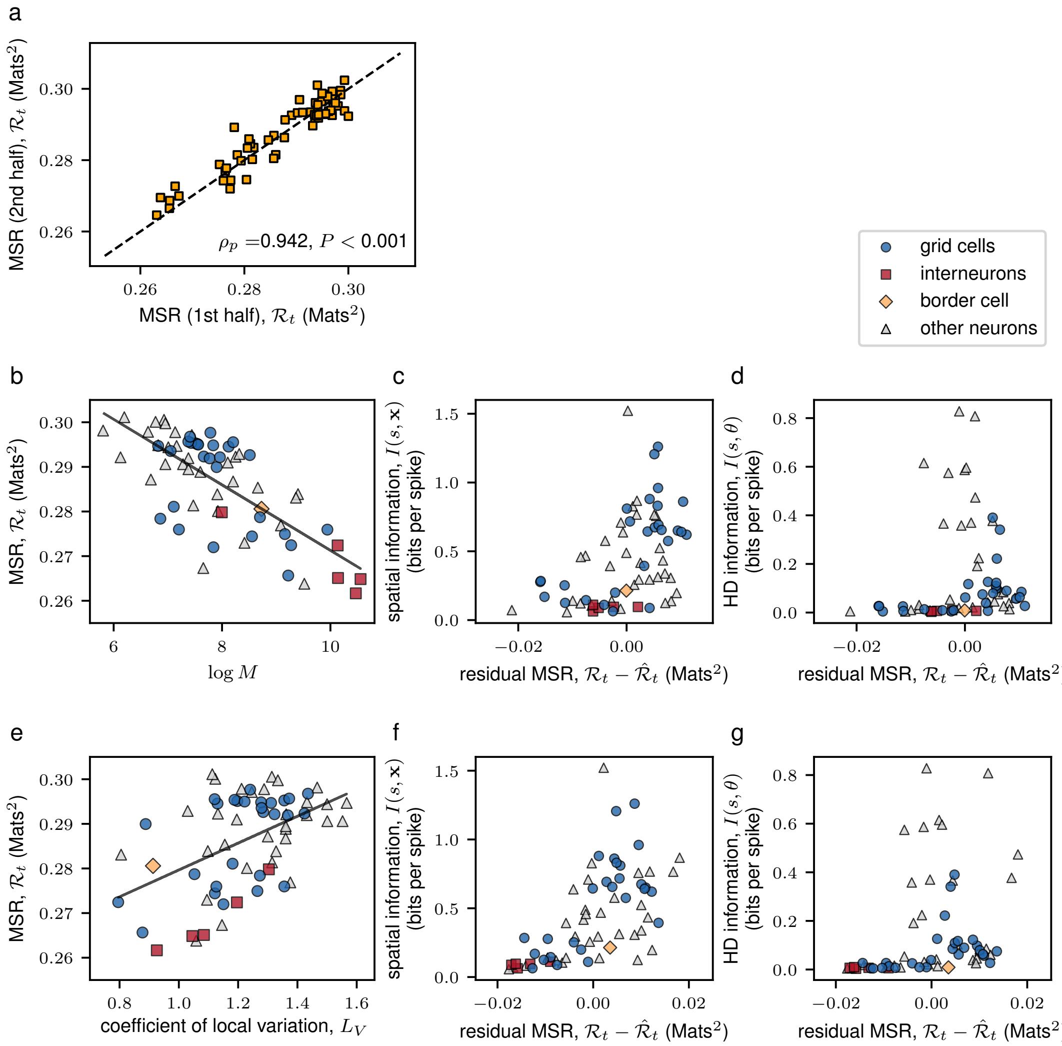

FIGURE S2. **The MSR is a robust measure and contains information beyond what the number of spikes and local variations can explain.** For each neuron, the MSR was calculated using only the first half and only the second half of the data (panel **a**). The scatter plot reports the two results. The linearity of the relationship between the two sets of partial data is quantified by the Pearson correlation ρ_p along with its P-value. The black dashed line indicates the linear fit. For the neurons in the mEC dataset, the MSR was linearly regressed with $\log M$ (panel **b**). The residual MSR, defined as the deviation of the MSR from the black regression line, were then correlated against spatial (panel **c**) and HD (panel **d**) information. The MSR was also linearly regressed with the coefficient of local variation, L_V (panel **e**). The residual MSRs were then correlated against spatial (panel **f**) and HD (panel **g**) information.

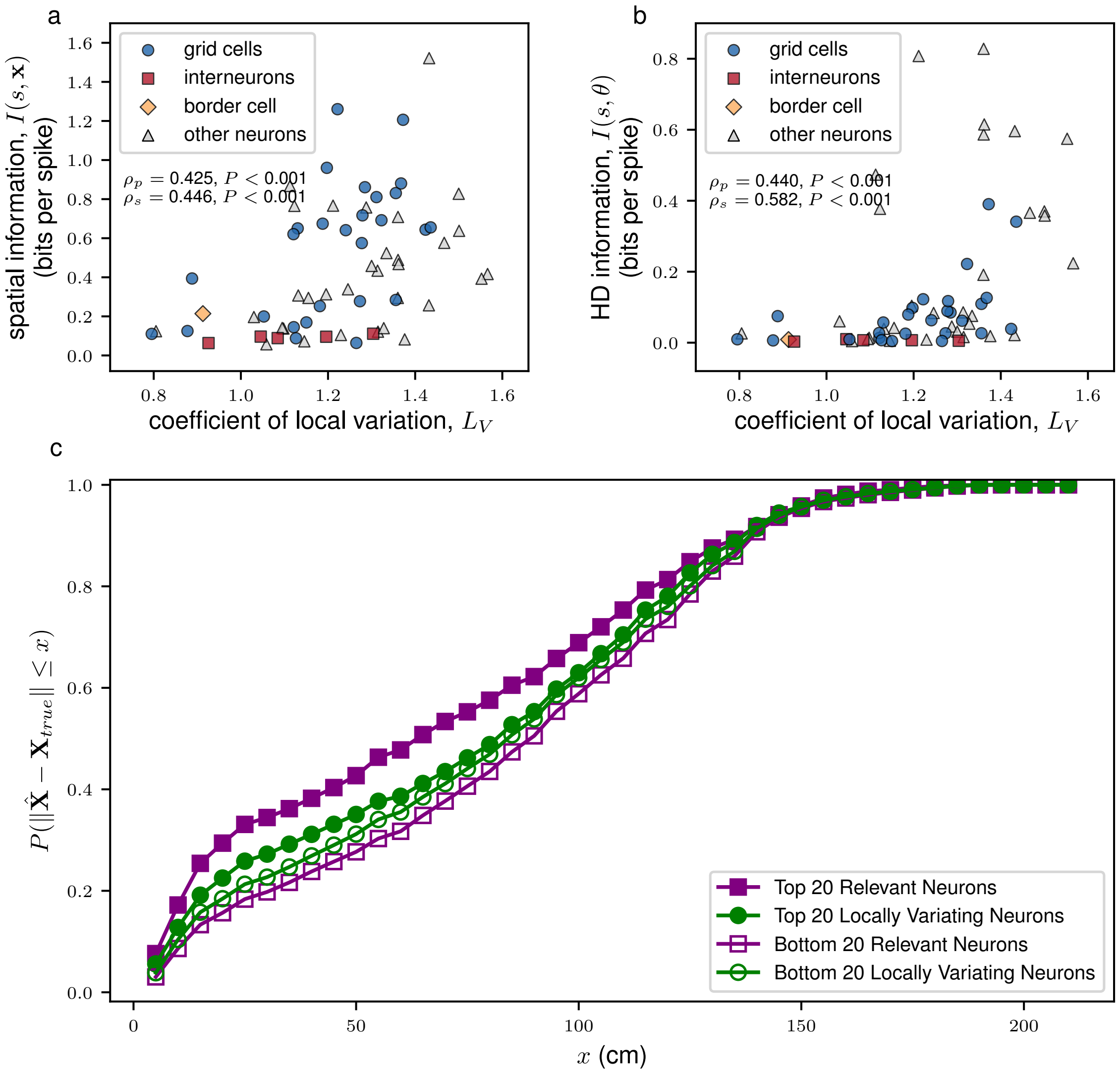

FIGURE S3. **Local variations in the interspike intervals can capture spatial and HD information but not decodable spatial information.** A scatter plot of the coefficient of local variation L_V vs. the spatial (HD) information is shown in **a** (**b**). The shapes of the scatter points indicate the identity of the neuron according to Stensola et al. (2012). The linearity and monotonicity of the multiscale relevance and the information measures were assessed by the Pearson's correlation, ρ_p, and the Spearman's correlation, ρ_s, respectively. The 20 top and bottom locally variating neurons (LVNs) were then used to decode position (See Main Text Section 5.6). Panel **c** shows the cumulative distribution of the decoding error, $\|\hat{\mathbf{X}} - \mathbf{X}_{true}\|$, for the RNs (solid violet squares) and LVNs (solid green circles) neurons as well as for the non-RNs (dashed violet squares) and non-LVNs (dashed green circles). In all the decoding procedures, time points where all the neurons in the ensemble was silent were discarded in the decoding process.

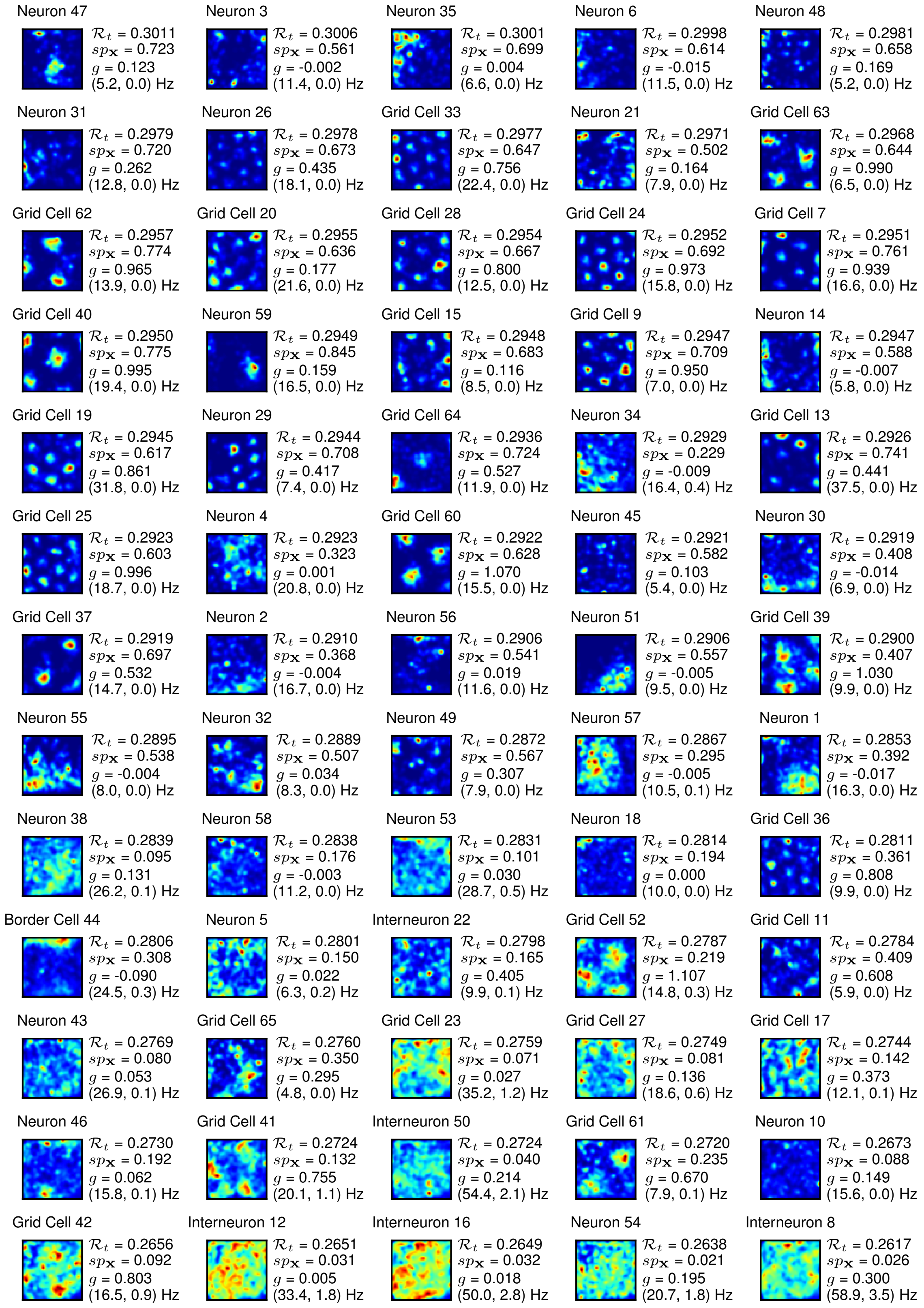

FIGURE S4. **RNs in the mEC exhibit spatially selective firing compared to non-RNs.** The spatial firing rate maps of the 65 neurons in the mEC data, sorted according to their MSR scores, are shown together with the calculated spatial sparsity, $sp_{\mathbf{x}}$, the grid score, g, and the maximum and minimum firing.

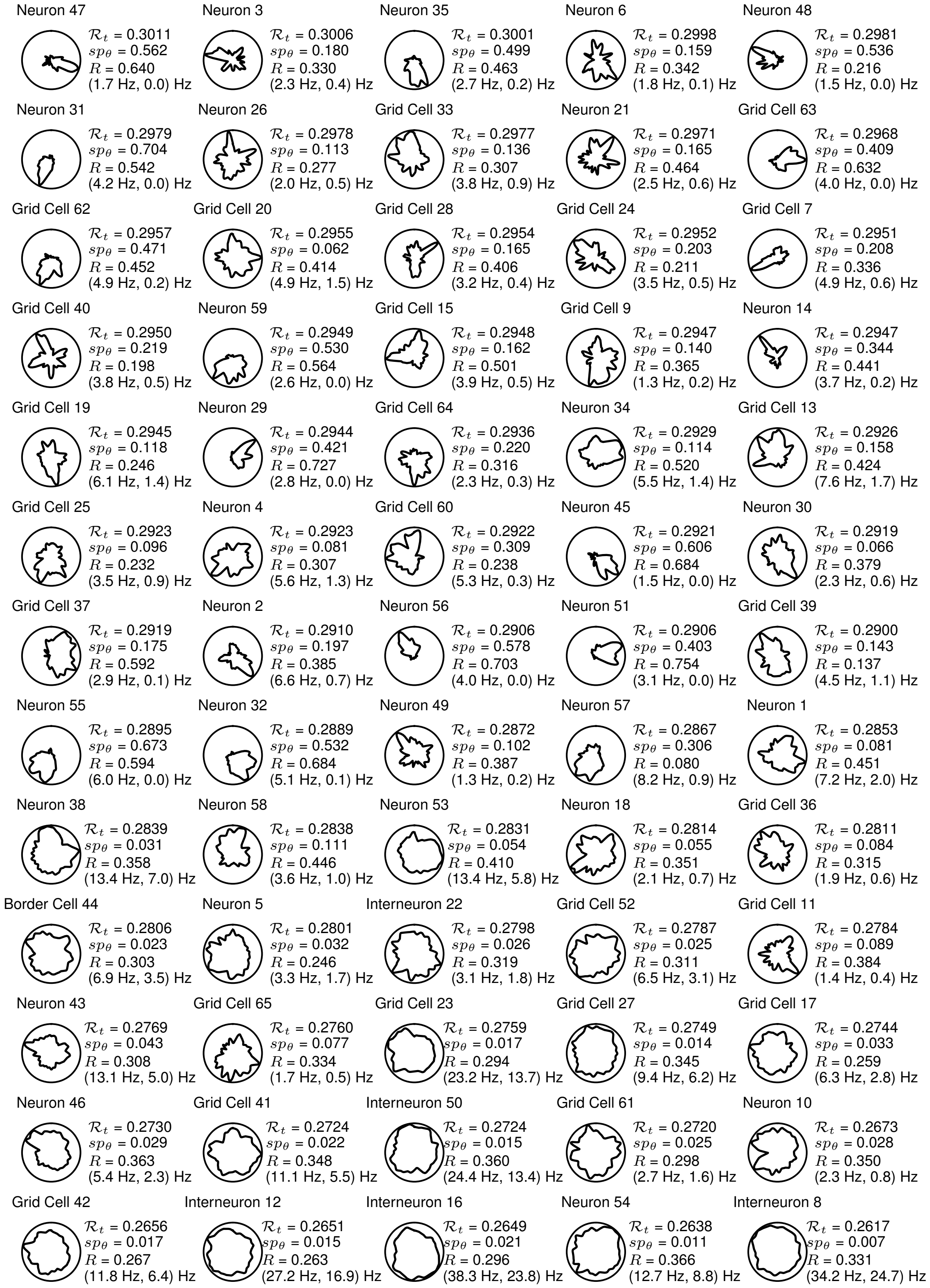

FIGURE S5. **RNs in the mEC exhibit HD selective firing compared to non-RNs.** The HD tuning curves of the 65 neurons in the mEC data, sorted according to their MSR scores, are shown together with the calculated HD sparsity, sp_θ, the Rayleigh mean vector length, R, and the maximum and minimum firing.

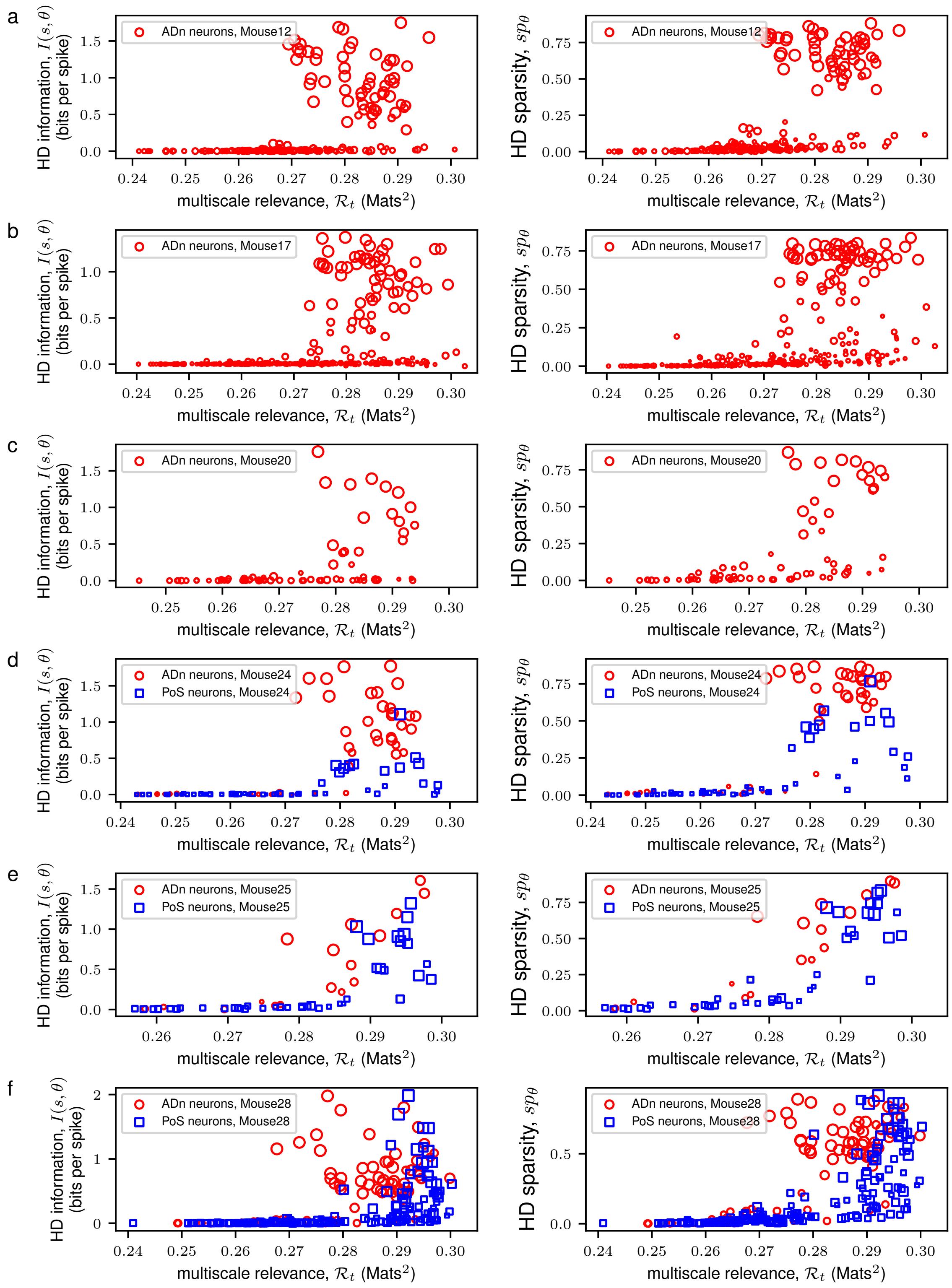

FIGURE S6. **MSR of neurons from the anterodorsal thalamic nucleus (ADn) and post-subicular (PoS) regions of 6 freely-behaving mice pooled from multiple recording sessions**. For each mouse, the MSR of the recorded neurons which had more than 100 recorded spikes in a session were calculated. The corresponding the HD information and sparsity (in bits per spike, see Main Text Section 5.4: Information, Sparsity and other Scores) were also calculated. ADn neurons are depicted in red circles while PoS neurons in blue squares. The size of each point reflects the mean vector lengths of the neurons wherein larger points indicate a unimodal distribution in the calculated HD tuning curves.

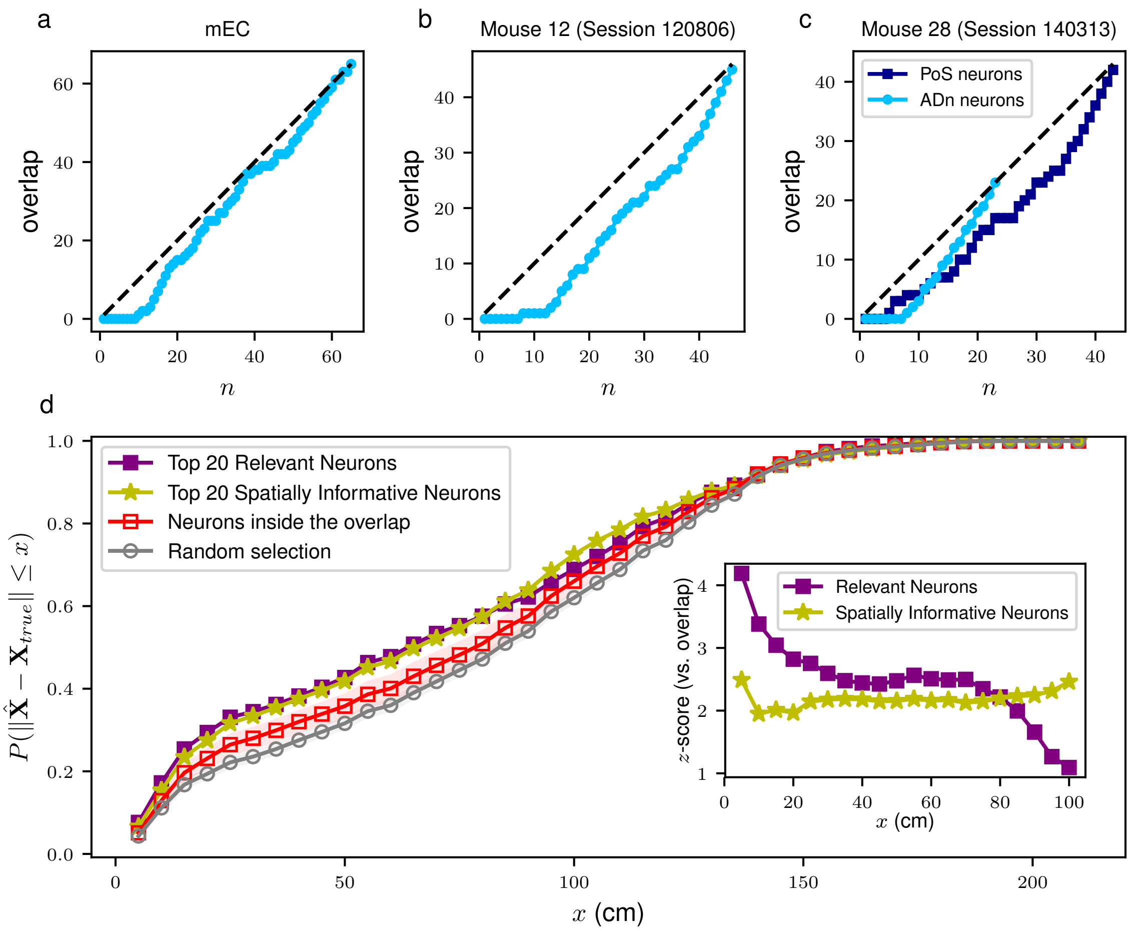

FIGURE S7. **The mEC neurons that were both RNs and INs do not contain the bulk of the decodable spatial information.** The overlap between the set of RNs and INs as a function of the size, n, of each set for the mEC (**a**), for the ADn of Mouse 12 (Session 120806) (**b**) and for the ADn and PoS of Mouse 28 (Session 140313) (**c**). To show how much decodable spatial information there is in the overlap between the RNs and spatial INs in the mEC at $n = 20$, we took the 14 overlapping neurons (ONs) and randomly chose 6 neurons outside of this overlap and performed a Bayesian positional decoding (see Main Text Section 5.6). The mean and standard errors of the cumulative distribution of decoding errors, $\|\hat{\mathbf{X}} - \mathbf{X}_{true}\|$, of the 14 ONs + 6 random neurons (n = 100 realizations) are shown in grey (**D**) together with the cumulative distribution of decoding errors of the RNs (violet squares) and spatial INs (yellow stars). For a given position error, x, a z-score can be calculated by measuring how many standard errors from the mean of the decoding errors for ONs is the decoding error of the RNs or of the spatial INs. These z-scores are shown in the inset of panel **d**.

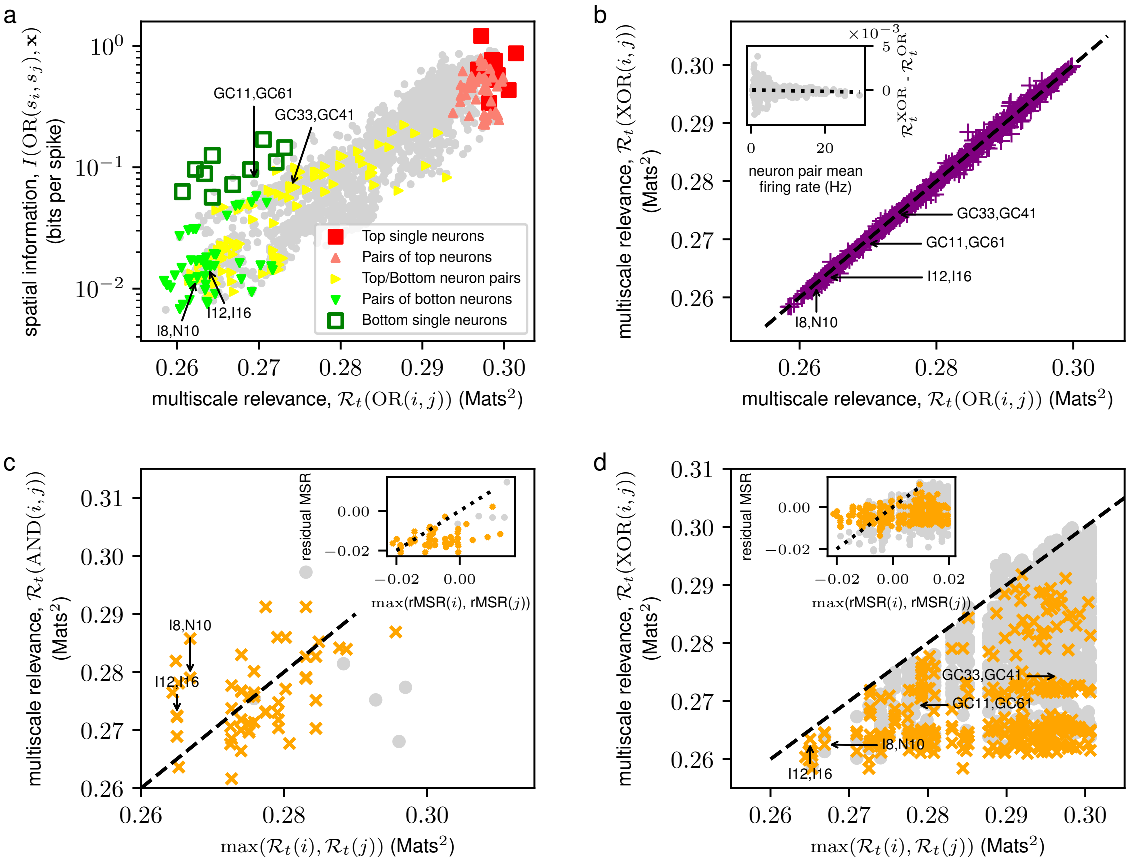

FIGURE S8. **Multiscale relevance (MSR) for spike trains built from AND, OR and XOR Boolean functions between pairs of neurons.** To study the possible application of MSR to neuronal ensembles, MSRs were calculated from spike trains resulting from AND, OR and XOR Boolean function between pairs of neurons (See Section 5.7). A scatter plot between the spatial information and the calculated MSR for spike trains constructed from OR Boolean function is shown in **a**. Red squares indicate the 10 single neurons with the highest MSR (top single neurons), green squares the 10 single neurons with the lowest MSRs (bottom single neurons), red circles the OR Boolean function from pairs of top neurons, green circles the pairs of bottom neurons, and yellow circles the pairs of top and bottom neurons. Similar results to panel **a** hold for HD information (plot not shown). Panel **b** shows a scatter plot between MSRs from OR Boolean function and from XOR Boolean function. The inset scatter plot show the difference between the MSRs from XOR and from OR Boolean function as a function of the average firing rate of the neuron pairs along with the best-fit line (dashed line). The comparison between the MSR of the OR and the XOR potentially allows us to probe the effects of common driving of the neurons, because the latter includes also simultaneous firing, whereas the former does not. We find that the MSR of the XOR is very similar to that of the OR. One way to interpret this lack of difference is that the role of common input to correlated firing is small. The lack of difference could also be due to small firing rates of neurons but given that we see the similarity between the MSR of the OR and XOR for almost all pairs makes this unlikely. Panel **c** (**d**) shows a scatter plot between the MSRs from an AND (XOR) Boolean function between pairs of neurons and the maximal MSR between the corresponding pairs. Residual MSRs are calculated by subtracting the part of the MSR that is explained by $\log M$ through linear regression. The corresponding inset scatter plots show a scatter plot between residual MSRs from pairs of neurons and the maximum residual MSR of the corresponding pairs. Orange crosses indicate neurons that are paired with an interneuron. The resulting MSR (of the OR or of the XOR) is almost always lower (and never significantly higher) than the MSR of the most relevant of the two neurons, suggests that individual neurons contain non-redundant information. Furthermore, for those pairs of neurons where we get a significant signal, in most of the cases, one of the neurons turns out to be an interneuron which suggests that interneurons play a peculiar role in the information aggregation. The neuron pairs considered in Supplementary Materials Fig. S9 are highlighted. GC: grid cell, I: interneuron, N: unclassified neuron.

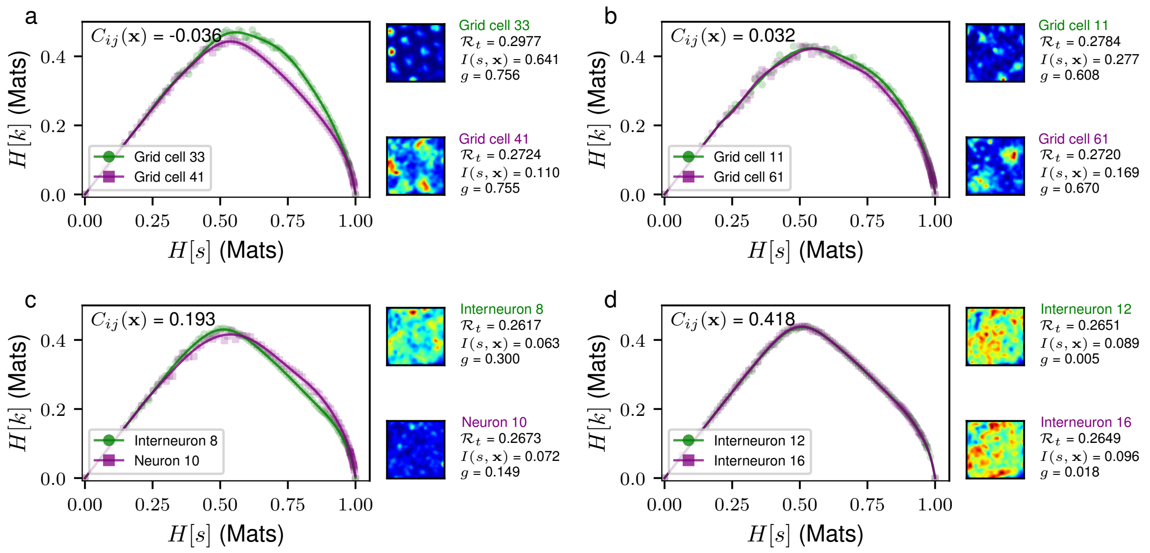

FIGURE S9. **Resolution vs. relevance curves for different pairs of neurons.** Panels show the resolution, $H[s]$, vs relevance $H[k]$ curves traced by different pairs of neurons: grid cells with high and low MSR (**a**), grid cells with low MSRs having similar grid scores (**b**), a neuron having a low MSR and an interneuron (**c**) and two interneurons (**d**). Each point, $(H[s], H[K])$, in this curve corresponds to a fixed binning time, Δt, with which we see the corresponding spike count codes. The spatial noise correlation, $C_{ij}(\mathbf{x})$ (see main text, section 5.8), for the neuron pairs are also indicated. The multiscale relevance of the boolean functions that can be constructed from these neuron pairs are indicated in Supplementary Materials Fig. S8.

Text S1: Relation between MSR and other measures of temporal structure

Characterizing the neural spiking can be done by studying the distribution of the time intervals between two succeeding spikes, known in literature as the interspike interval (ISI) distribution which allows us to see whether a neuron fires in bursts (Ebbesen et al., 2016; Sharp and Green, 1994). Note that given the time stamps of neural activity $\{t_1, \ldots, t_M\}$, the interspike interval is given by $\{\tau_1, \ldots, \tau_{M-1}\}$ where $\tau_i = t_{i+1} - t_i$. Because the multi-scale relevance (MSR) is built to separate relevant neurons from the irrelevant ones through their temporal structures in the neural spiking, we wanted to assess how the proposed measure scales with the characteristics that give structure to temporal events. In the context of the temporal activity of a neuron, a feature of the relevance measure, $H[K]$ is that highly regular, equally-spaced ISI are attributed with a low measure. On the other hand, ISI that follow broad, non-trivial distributions are attributed with a high relevance measure. Hence, we expected that the relevance measure, and therefore the MSR, captures non-trivial bursty patterns of neurons.

To study how MSR behaves with respect to the characteristics of ISI, we considered a stretched exponential distribution

$$P^u_{SE}(\tau) = \frac{u}{\tau_0}\left[\frac{\tau}{\tau_0}\right]^{u-1} \exp\left[-\left(\frac{\tau}{\tau_0}\right)^u\right] \tag{S1}$$

with which the parameter u allows us to define the broadness of underlying distribution and τ_0 is the characteristic time constant of the random event. For Poisson processes, the ISI follow an exponential distribution corresponding to $u = 1$ in Eq. (S1). For $u < 1$, the ISI distribution becomes broad and tends to a power law distribution with an exponent of -1 in the limit when $u \to 0$. On the other hand, for $u > 1$, the distribution becomes narrower and tends to a Dirac delta function in the limit when $u \to \infty$.

Upon fixing the parameters u and τ_0 which fixes the stretched exponential distribution in Eq. (S1), random ISI, τ_i, could then be sampled independently from Eq. (S1) so as to generate a time series of 100,000 time units. The MSRs of each time series could then be calculated using the methods described in the main text (Section 2).

To characterize the temporal structures of both the simulated data and neural data, we adapted the measures of bursty-ness and memory of Goh and Barabasi (Goh and Barabási, 2008). While the bursty-ness coefficient, b defined as

$$b = \frac{\sigma_\tau - \mu_\tau}{\sigma_\tau + \mu_\tau}, \tag{S2}$$

measures the broadness of the underlying ISI distribution with μ_τ and σ_τ as the mean and standard deviations of the ISI respectively, the memory coefficient, m defined as

$$m = \frac{1}{M-2}\sum_{j=1}^{M-2}\frac{(\tau_j - \mu_\tau)(\tau_{j+1} - \mu_\tau)}{\sigma_\tau^2}, \tag{S3}$$

measures the short-time correlation between events.

For the stretched exponential distribution in Eq. (S1), the mean and standard deviations could be computed as

$$\mu_\tau = \tau_0 \Gamma\left(\frac{u+1}{u}\right) \tag{S4}$$

and

$$\sigma_\tau = \tau_0 \sqrt{\Gamma\left(\frac{u+2}{u}\right) - \Gamma\left(\frac{u+1}{u}\right)^2} \tag{S5}$$

where $\Gamma(x) \equiv (x-1)!$ is the gamma function. With these closed-form relationships, we could now study the limiting properties of the burstiness and memory coefficients. For Poisson processes, the mean, μ_τ, and standard deviation, σ_τ, coincide, i.e. $\mu_\tau = \sigma_\tau = \tau_0$, and thus with Eq. (S2), give $b = 0$. For broad distributions, $u < 1$ in Eq. (S1), $\sigma_\tau > \mu_\tau$ which gives $b > 0$ and tends to approach $b \to 1$ in the limit $u \to 0$. On the other hand, for narrow distributions, $u > 1$ in Eq. (S1), $\sigma_\tau < \mu_\tau$ resulting to $b < 0$ and tends to $b \to -1$ in the limit $u \to \infty$. Hence, the bursty-ness parameter, b, is a bounded parameter, i.e., $b \in [-1, 1]$.

For the synthetic datasets, note that fixing the parameter u automatically fixes the burstyness coefficient, b. However, because the synthetic ISI are sampled independently, the memory coefficient, m, is approximately zero. Short-term memory can then be introduced by first sorting the ISI in decreasing (or increasing) order which results to $m \approx 1$. Randomly shuffling a subset of the ordered ISI (100 events at a time in this case) results to a monotonic decrease of m. In the limit of infinite data, the memory coefficient is bounded by $[-1, 1]$. These bounds may no longer hold in the case of limited data. Despite this, a positive memory coefficient indicates that a short (long) ISI between events tends to be followed by another short (long) interval and a negative memory coefficient indicates that a short (long) ISI between events tends to be followed by a long (short) interval.

With this, we found that the MSR increased with bursty-ness and memory for the synthetically generated dataset as seen in Supplementary Materials Fig. S1a and b. We also sought to characterize the relationship between the number of events, M, with the MSR which can be addressed by changing the characteristic time constant, τ_0, in Eq. (S1) wherein decreasing τ_0 leads to more events and thus, increased $\log M$. We found that MSR decreased with $\log M$ as seen in Supplementary Materials Fig. S1c. This result is indicative that MSR of randomly generated events can be explained by $\log M$.

Since the MSR is constructed as a measure of dynamical variablity, we compared our results on synthetically generated datasets with the coefficient of local variation, L_V, (Shinomoto et al., 2005, 2003, 2009) defined as

$$L_V = \frac{1}{M-1} \sum_{j=1}^{M-1} \frac{3\left(\tau_j - \tau_{j+1}\right)^2}{\left(\tau_j + \tau_{j+1}\right)^2} \tag{1}$$

where the factor 3 in the summand was taken such that, for a Poisson process, $L_V = 1$. With this, we found that the L_V increases with increasing bursty-ness coefficient, b, indicating that power law ISI distributions lead to highly locally variating spiking events (see Supplementary Materials Fig. S1d). Also, we found that the L_V decreases with increasing short-term memory, m (see Supplementary Materials Fig. S1e). Finally, like the MSR, we also found a dependence of the L_V with the $\log M$ (see Supplementary Materials Fig. S1f).

Following the results on synthetic data, we also analyzed temporal characteristics in real neural dataset. In the case of neurons in the mEC data, we also found that MSR decreased with the logarithm of the number of observed spikes, $\log M$, as shown in Supplementary Materials Fig. S2b. To determine how much of the calculated MSRs can be explained by the number of observed spikes, M, we linearly regressed MSR with $\log M$ shown as the dashed line in Supplementary Materials Fig. S2b. Residuals were then calculated as the deviation of the calculated MSR from the regression line and thus, captures the amount of

MSR that cannot be explained by $\log M$ alone. We showed in Supplementary Materials Fig. S2c and d that the MSR for real dataset still contained information going beyond $\log M$ as the residual MSRs (with respect to $\log M$) still retained the dependence with spatial and HD information as already observed in the main text (Fig. 2). We also observed a positive correlation between MSR and L_V. However, through residual analysis, we found that the residual MSRs (with respect to L_V) still contained spatial and HD information as seen in Supplementary Materials Fig. S2f and g.

References

Ebbesen, C. L., Reifenstein, E. T., Tang, Q., Burgalossi, A., Ray, S., Schreiber, S., Kempter, R., and Brecht, M. (2016). Cell type-specific differences in spike timing and spike shape in the rat parasubiculum and superficial medial entorhinal cortex. *Cell reports*, 16(4):1005–1015.

Goh, K.-I. and Barabási, A.-L. (2008). Burstiness and memory in complex systems. *EPL (Europhysics Letters)*, 81(4):48002.

Sharp, P. E. and Green, C. (1994). Spatial correlates of firing patterns of single cells in the subiculum of the freely moving rat. *Journal of Neuroscience*, 14(4):2339–2356.

Shinomoto, S., Kim, H., Shimokawa, T., Matsuno, N., Funahashi, S., Shima, K., Fujita, I., Tamura, H., Doi, T., Kawano, K., et al. (2009). Relating neuronal firing patterns to functional differentiation of cerebral cortex. *PLoS computational biology*, 5(7):e1000433.

Shinomoto, S., Miura, K., and Koyama, S. (2005). A measure of local variation of interspike intervals. *Biosystems*, 79(1-3):67–72.

Shinomoto, S., Shima, K., and Tanji, J. (2003). Differences in spiking patterns among cortical neurons. *Neural Computation*, 15(12):2823–2842.

Stensola, H., Stensola, T., Solstad, T., Frøland, K., Moser, M.-B., and Moser, E. I. (2012). The entorhinal grid map is discretized. *Nature*, 492(7427):72–78.
